# Absolute quantification of the living skin microbiome overcomes relic-DNA bias and reveals specific patterns across volunteers

**DOI:** 10.1186/s40168-025-02063-4

**Published:** 2025-03-04

**Authors:** Deepan Thiruppathy, Oriane Moyne, Clarisse Marotz, Michael Williams, Perris Navarro, Livia Zaramela, Karsten Zengler

**Affiliations:** 1 Department of Bioengineering, University of California San Diego, La Jolla, San Diego, CA 92093 USA; 2 Department of Pediatrics, School of Medicine, University of California San Diego, La Jolla, San Diego, CA 92093 USA; 3 Center for Microbiome Innovation, University of California San Diego, La Jolla, San Diego, CA 92093 USA; 4 Program in Materials Science and Engineering, University of California San Diego, La Jolla, San Diego, CA 92093 USA

**Keywords:** Skin microbiome, Absolute abundance, Relic DNA, Metagenomics, Flow cytometry

## Abstract

**Background:**

As the first line of defense against external pathogens, the skin and its resident microbiota are responsible for protection and eubiosis. Innovations in DNA sequencing have significantly increased our knowledge of the skin microbiome. However, current characterizations do not discriminate between DNA from live cells and remnant DNA from dead organisms (relic DNA), resulting in a combined readout of all microorganisms that were and are currently present on the skin rather than the actual living population of the microbiome. Additionally, most methods lack the capability for absolute quantification of the microbial load on the skin, complicating the extrapolation of clinically relevant information.

**Results:**

Here, we integrated relic-DNA depletion with shotgun metagenomics and bacterial load determination to quantify live bacterial cell abundances across different skin sites. Though we discovered up to 90% of microbial DNA from the skin to be relic DNA, we saw no significant effect of this on the relative abundances of taxa determined by shotgun sequencing. Relic-DNA depletion prior to sequencing strengthened underlying patterns between microbiomes across volunteers and reduced intraindividual similarity. We determined the absolute abundance and the fraction of population alive for several common skin taxa across body sites and found taxa-specific differential abundance of live bacteria across regions to be different from estimates generated by total DNA (live + dead) sequencing.

**Conclusions:**

Our results reveal the significant bias relic DNA has on the quantification of low biomass samples like the skin. The reduced intraindividual similarity across samples following relic-DNA depletion highlights the bias introduced by traditional (total DNA) sequencing in diversity comparisons across samples. The divergent levels of cell viability measured across different skin sites, along with the inconsistencies in taxa differential abundance determined by total vs live cell DNA sequencing, suggest an important hypothesis for certain sites being susceptible to pathogen infection. Overall, our study demonstrates a characterization of the skin microbiome that overcomes relic-DNA bias to provide a baseline for live microbiota that will further improve mechanistic studies of infection, disease progression, and the design of therapies for the skin.

Video Abstract

**Supplementary Information:**

The online version contains supplementary material available at 10.1186/s40168-025-02063-4.

## Introduction

The skin serves as a crucial barrier against pathogens and moisture loss [1–3]. More than a simple physical barrier, it is also a complex and spatially structured ecosystem that hosts a diverse array of microorganisms across various physiological and topographical niches [4, 5]. Research on skin microbes has evolved from culture-based methods [6, 7], to culture-independent sequencing techniques, revealing the skin microbiota’s unique characteristics [8–11].

Due to harsh environmental conditions and nutrient limitation on the skin’s surface, the skin microbiome is characterized by low microbial biomass [12], complicating comprehensive culture-based studies and affecting the quality of studies based on marker genes, i.e., amplicon sequencing [13, 14]. While shotgun metagenomic sequencing with increased coverage and improved DNA extraction protocols can partially mitigate these issues [15, 16], the stability and prolonged persistence of extracellular DNA [17, 18] can result in a significant portion of the sequenced DNA originating from dead microbial cells, so-called relic DNA. Consequently, most metagenomic studies face challenges distinguishing between relic DNA and DNA from live, intact bacterial cells that interact functionally with the host and influence disease [19].

Most sequencing-based studies are compositional, and the majority currently rely on relative abundance data. This introduces a mathematical bias [20, 21], where an observed increase in one taxon’s relative abundance must coincide with a decrease in another’s, regardless of whether the total bacterial cell density has changed. Though several methods have been developed to counter this bias (e.g., DNA spike-ins prior to sequencing [22, 23] or differential ranking post-sequencing [24]), microbiome characterization is still susceptible to bias from relic DNA, especially if its proportion in the sample is high compared to intact-cell DNA [25]. Because of its low biomass and frequent exposure to external microorganisms, skin swab samples can contain a significant proportion of relic DNA potentially misrepresenting the viable cell population of the skin microbiome [26].

**Fig. 1:**
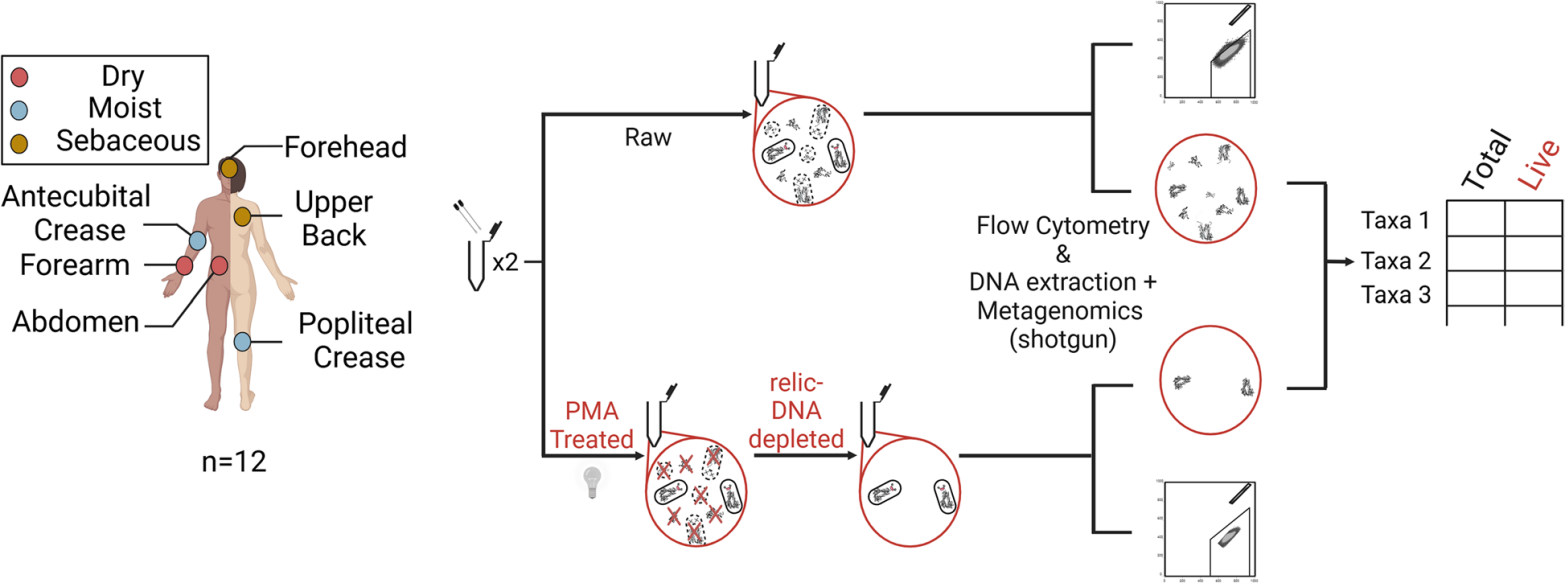
Study design. Twelve sets of samples from volunteers across six different body sites were collected. Each sample was collected in duplicate and processed in parallel, with one swab being submitted to PMA treatment to eliminate signal from dead cells, while the other swab was left untreated. Both duplicates were used for cell quantification by flow cytometry and metagenomic characterization

Current estimates of microbial biomass on the skin range from 1e + 4 to 1e + 6 cells per square centimeter [27, 28]; however, they do not discriminate between live and dead members. On the other hand, culture-dependent methods [29, 30] are limited to detecting only the microbes that can form colonies in the laboratory [31], whereas culture-independent methods [26, 27] can be biased by primer selection and the quality of DNA collected [32, 33]; as a result, the actual population of live bacteria on the skin is currently unknown. One method that has been deployed to counter this issue in several different microbiome systems is the treatment of samples with an intact-cell-membrane impermeable dye called propidium monoazide (PMA) prior to characterization using DNA amplification methods. PMA exclusively binds covalently to DNA that is exposed and unprotected by an intact cell wall or membrane upon light activation, effectively cross-linking it. Visible light causes the azide group of the PMA molecule to be photolytically cleaved and form a covalent bond with DNA [34], which fragments the DNA, subsequently eliminating any exposed DNA from downstream analysis [35]. Any excess PMA in the sample reacts with water and becomes inert. This cross-linking renders the DNA insoluble and non-amplifiable, as it prevents the DNA polymerase from accessing or replicating the modified regions [36]. Additionally, PMA crosslinking blocks the binding of DNA intercalators like SYBR, making the modified DNA undetectable in fluorescence-based assays such as in situ hybridization or flow cytometry. This selective mechanism allows PMA to differentiate live cells with intact membranes from dead cells with compromised membranes, i.e., relic DNA. This selective process has been applied to amplicon-based sequencing to identify viable bacterial populations in diverse sample types, including soil, wastewater, digesters, seawater, and gut [34, 37–40], as well as in low microbial biomass samples like saliva [41, 42] and samples with significant human contamination such as sputum [43]. Amplicon-based approaches using universal 16S rRNA primers coupled with PMA treatment have revealed that relic DNA is abundant on the skin surface and can lead to an overrepresentation of taxa identified via sequencing [26]. In a previous study, we combined PMA treatment of saliva with flow cytometry to quantify relic-DNA bias in amplicon sequencing and biomass estimates of the oral microbiome [44]. Here, we used shotgun metagenomic sequencing with PMA treatment of skin swabs for a comprehensive assessment of the microbiome that addresses amplicon-based underrepresentation of sample diversity and attains resolution at the species level [45–47]. We combined this approach with flow cytometry for both untreated (raw) and PMA-treated skin swab samples to characterize and quantify both the total and the live cell abundance across skin sites representing distinct microenvironments (Fig. 1). This approach allowed us to assess relic-DNA bias in shotgun metagenomics of skin swabs, to reveal biological patterns after relic-DNA depletion, and to quantify live-cell abundances of bacterial taxa residing on the skin surface. Notably, low counts of live skin commensals could play a role in fighting off pathogens and thus might affect skin disease and infection, an aspect that can be evaluated in future studies using an approach as described here.

## Materials and methods

### Study cohort

Volunteers were recruited in accordance with the institutional review board (IRB) protocol number 150275. Participant demographics are detailed in the supplementary metadata files. Eleven (11) human participants were recruited for the study and were swabbed on defined areas of the skin at 6 different body sites as detailed in Fig. 1. Volunteer 1 was swabbed a second time, amounting to 12 sets of samples in total. Participants were instructed not to wash the selected regions, no excessive workouts, and not to use cosmetics or lotions at least 12 h prior to sample collection. A short metadata questionnaire was confidentially filled in by each participant.

### Sample collection

Plastic patterns were used to standardize the sampling area for each body site between patients (Table S2). Each site was swabbed with two sterile plastic swabs (Puritan, cat. no. 3406-H) soaked in 1 × PBS simultaneously using a consistent technique. The swab head was gradually rotated while rubbing across each site for 30 s. Swab heads were broken off into Eppendorf tubes. Swabs were immediately vortexed for 2 min at max speed. The swab head was then removed from solution using sterile forceps while gently pressing the head against the inside of the tube to drain all liquid from it. The solution was then filtered in 5-µm filter (TISCH, cat. no. SF18200) to remove human cells and swab head plastic debris dislodged during vortexing. The two tubes per swab site were pooled together and gently mixed. A total of 400 µL from this pool were transferred to a separate tube for PMA treatment. All volunteers were swabbed by the same individual to maintain consistency across samples. Each sample underwent parallel processing for PMA treatment and untreated controls, followed by quantification with flow cytometry, and was stored at − 80 °C on the same day to avoid bias in cell counts caused by freeze–thaw cycles. To ensure all steps up to freezing could be completed for each volunteer’s swabs, the number of volunteers sampled per day was limited to three.

### PMA treatment for relic-DNA depletion

Four microliters of 100-µM PMA (Biotium, cat. no. 40013) was added to 400 µL of bacterial extract from the skin swabs (1-µM PMA final). Samples were then briefly vortexed and incubated in the dark at room temperature for 5 min. The samples were then laid horizontally on ice 20 cm from direct light source (488 nm) for 25 min. During this period, the PMA covalently binds to the relic DNA and cross-links it. To ensure even distribution of the PMA molecules, the samples were gently vortexed (speed level 3 on the Vortex-Genie 2 Lab Genie) every 5 min during this period. At the same time, untreated (raw) versions of the sample were stored in saline solution on ice in the dark.

### Flow cytometry

A 250-µL aliquot of both PMA-treated and non-PMA-treated samples were used for absolute microbiome quantification by flow cytometry. Briefly, 1.25 µL of 20 × SYBR Green (SYBR™ Green I Nucleic Acid Gel Stain, cat. no. S7563) was added to the samples (final SYBR concentration of 0.1 ×), and tubes were incubated in the dark for exactly 15 min. Thirty microliters of AccuCount fluorescent particles (ACFP-70–10, Spherotech) fluorescent sorting beads (mixed well) was then added to the samples and mixed by vortexing. Samples were processed on a SH800 Cell Sorter (Sony Biotechnology) using a 100-µm chip with the threshold set on the FL1 at 0.06% and gain settings as FSC (forward scatter) = 4 and BSC (side scatter) = 25%. The gain settings were set following prior studies by Props et al. (2018) and Prest et al. (2013) [13, 48]. Briefly, the settings were set using AccuCount fluorescent particles (ACFP-70–10; Spherotech) with FSC set to the lowest gain to avoid detector saturation, and BSC was attenuated to the highest level to capture the weak signal from low-granular microbial cells. Gain settings were lowered for FSC and raised for BSC until the beads appeared distinct from the background (and clustered on the lower right panel of the FL1 vs FL4 plot). Gain settings for FL1 (channel 1 (green) for SYBR emission detection) = 43% and FL4 (channel 2 (red) for detecting background signals) = 50% were set using a mix of unstained and stained beads. The gating strategy was adapted from our earlier study [44], where, first, the fluorescent microbial cells were gated from the background on an FL1–FL4 density plot as those with a high FSC and low SSC signal (lower right quadrant, Fig. S8a). Aggregates were excluded by taking the linear fraction on a graph of area (FL1-A) versus height (FL1-H) of the FL1 signal (upper left quadrant, Fig. S8b). Large events detected on the forward scatter (FSC) versus side scatter (BSC) plot were removed (upper left quadrant, Fig. S8c). Sterile phosphate-buffered saline (PBS) (sheath fluid for flow cytometer) was run between samples to reduce cross contamination. Final cell counts per microliter calculations were performed following manufacturer’s instructions of the AccuCount counting beads. Total cells per square centimeter from the sampled site were computed by multiplying the estimated concentration by the volume of the sample run (250 µL) with the swabbed site area (Table S2).

### DNA extraction and library preparation

DNA was extracted from a 150-µL aliquot of both PMA-treated and non-PMA-treated samples using the DNeasy PowerSoil Pro Kit (Qiagen, cat. no. 47016). The concentration of extracted DNA was measured using a Qubit dsDNA HS Assay Kit (Invitrogen, cat. no. Q33231) and a QuBit 2.0 Fluorometer (Invitrogen). DNA-seq libraries were prepared for samples using 1-ng DNA input following the Nextera XT library preparation kit protocol (Ilumina, cat. no. FC-131–1096). SYBR Green was added to the libraries to follow amplification in real time, and samples that amplified were sequenced.

### Shotgun sequencing metagenomics

DNA-seq libraries were quality-checked for integrity, and their average size was measured using a 4200 TapeStation system (Agilent). If necessary, libraries were cleaned prior to sequencing using Select-a-Size DNA Clean & Concentrator (Zymo Research, cat. no. D4080). Library concentrations were measured using a Qubit dsDNA HS Assay Kit (Invitrogen, cat. no. Q33231) and a QuBit 2.0 Fluorometer (Invitrogen) and pooled to equal amounts. DNA sequencing was performed on an Illumina NovaSeq 6000, PE100 platform. Controls were also sequenced from the swab only, the DNA extraction kit only, and the Nextera Library Prep Kit. For the swab control, a new swab was pre-moistened with 1 × PBS and aired for 30 s in the same room used for swabbing volunteers. For the kits control, molecular grade (DNAse/RNAse free) water was used as sample input.

### Data processing

Adapter sequences were trimmed from metagenomic sequencing data using TrimGalore (Cutadapt) version 1.18 [49] and quality controlled using FastQC version 0.11.9 [50]. Large bowtie2 indices were constructed after concatenating the trimmed and quality-filtered swab and blank and kit control samples. All experimental samples were first filtered for swab, blank, and kit control reads using bowtie2 version 2.3.2 [51] in –very-sensitive-local– mode to ensure strict filtering of direct, end-to-end matches. Reads that passed this filter were then filtered against the human genome (GRCh38.p14) using –very-sensitive-global– parameters to guarantee any partial matches to the human genome are also filtered. Mapping percent to human genome is shown in Fig. S1c. Filtered reads were then aligned to the Skin Microbiome Genomic Catalogue [8] database using –very-sensitive-local– parameters in bowtie2 and filtered for 60% genome coverage using Zebra filter [52] (see Fig. S1d for coverage density plots). Taxonomic read count tables were obtained using Woltka version 0.1.1 [53]. Read count tables were imported in R version 3.6.3 (R Core Team, 2020) and normalized to genome lengths and counts per million for statistical analyses (RPKM). For independent relic-DNA portion estimates, samples were aligned to the Web of Life database [54].

### Statistical analysis

All statistical analyses were performed in the R software. Data are represented as median (interquartile range Q3–Q1) unless otherwise indicated. For all box plots, black center lines represent the median and box edges the first and third quartiles. Spearman correlations (r) of nonzero values were used for all correlation coefficients. The nonparametric tests Wilcoxon rank-sum and Kruskal–Wallis were used to determine statistically significant differences between microbial populations, and to identify significant inter-category comparisons, we used a post hoc multiple comparison Dunn’s test. Unless otherwise indicated, *p*-values were adjusted for multiple comparisons using the p.adjust function in R using “FDR” method [55]. Statistical significance was ascribed to an alpha level of the adjusted *p*-values < 0.05 (***p < 0.005, **p < 0.01, *p < 0.05, ns non-significant). For core phylogenetic alpha-diversity metrics, the feature table was imported as a phyloseq object (phyloseq ver 1.38.0) [56], and the estimate_richness method from the vegan package (Vegan ver 2.6–4) [57] was used to compute “Shannon” and “Simpson” diversity metrics. For core phylogenetic beta-diversity metrics, we used robust Aitchison distance metric from the gemelli package [58] via the QIIME2 plugin interface [59].

## Results

### Study design

Sixty-six samples were collected from 6 body sites representing 3 skin types: sebaceous (forehead and upper back), moist (antecubital and popliteal creases), and dry (forearm and abdomen) (full list of metadata is provided as Tables S1 and S2). Swabs soaked in saline were used to ensure maximal and consistent microbial biomass collection with minimal invasion and minimal lysis of cells [16]. Samples were split and subject to treatment with or without PMA followed by flow cytometry sorting and subsequent metagenomic sequencing. This approach enabled us to differentiate skin microbiome signatures originating from live cells only versus total microbial cells (Fig. 1). Of the 144 total samples collected, 141 samples had detectable levels of bacterial cells after gating, and 123 samples had sufficient DNA for shotgun sequencing (see the “Materials and methods”).

## Characterizing the living population of the skin microbiome reveals stronger beta-diversity patterns across volunteers

Metagenomic sequencing was performed on untreated (raw) and PMA-treated samples (*n* = 123 samples). Data were processed, filtered against controls and the human genome, aligned to skin-specific [8] and general databases [54], and normalized for taxa genome lengths (see the “Materials and methods”). The principal component analysis (PCA) of raw and PMA-treated samples confirmed earlier findings that the sampled site’s microenvironment determines clustering (Fig. S2a, b) [28, 60, 61]. Species-level taxonomy of raw samples identified *Cutibacterium acnes* as the dominant taxon across all body sites, consistent with prior shotgun sequencing studies [8, 60, 61]. PMA-treated, relic-DNA-depleted samples showed no significant taxonomic differences (paired Wilcoxon test, FDR-corrected *p* > 0.05, *n* = 51), indicating relic DNA does not bias body-site or skin type characterization (Fig. S2c). PMA-dependent taxon-level changes were assessed using the PMA-index metric [26] for the top 15 taxa ranked by median RPKM across samples (see the “Materials and methods”). Most taxa had a PMA index near 0.5, suggesting minimal relic-DNA bias in compositional estimates (Fig. S3). Relic-DNA effects on alpha diversity and taxonomic evenness were assessed using Shannon diversity and Simpson evenness metrics. Both metrics were uncorrelated with sequencing depth (Spearman *r* = − 0.3 for both, Fig. S4a, b) and showed no significant differences between treatments across body sites. Raw and PMA-treated samples consistently showed lowest diversity scores in forehead sebaceous sites, while the popliteal crease (moist skin) had the highest evenness and Shannon diversity, aligning with current estimates [1, 62, 63] (Fig. S4c, d, e, f). Together, these findings indicate that relic DNA does not introduce taxon-specific bias in the skin microbiome, and diversity and evenness trends remain unaffected.

To address the significant sparsity characteristic of low-biomass microbiome datasets like the skin, we employed a weighted centered log-ratio transformation distance metric, robust Aitchison distance metric [58], to evaluate the effect of relic-DNA removal on inter- and intraindividual similarities. Robust Aitchison distance does not assume the data are dense and is robust to missing data by treating it as unobserved through an adaptation of centered log-ratio transformation of the data (RCLR) [64, 65]. We calculated the distance matrix on all, raw only and PMA-treated only samples (*n* = 123 samples, raw = 66, *PMA* = 67) and computed the permutational multivariate analysis of variance (PERMANOVA), pseudo-F statistic (F), and *p*-value to evaluate clustering by sample metadata (Table 1).

**Table 1:** Effect of the factor metadata on sample variability. Results of a PERMANOVA analysis computed on RPCA distances (see the “Materials and methods”)

Samples	Factor metadata	*F*-statistic	*p*-value
All (raw + PMA-treated)	Treatment	1.28	0.309 (ns)
Raw	Volunteer	16.95	0.001***
	Skin type	1.72	0.13 (ns)
	Body site	1.26	0.25 (ns)
	Sex	14.3	0.001***
PMA-treated	Volunteer	5.19	0.001***
	Skin type	12.02	0.001***
	Body site	4.9	0.001***
	Sex	7.34	0.001***

Paired Wilcoxon test

^***^p < 0.001

^**^p < 0.01, *p* > 0.05, *ns* non-significant

**Fig. 2:**
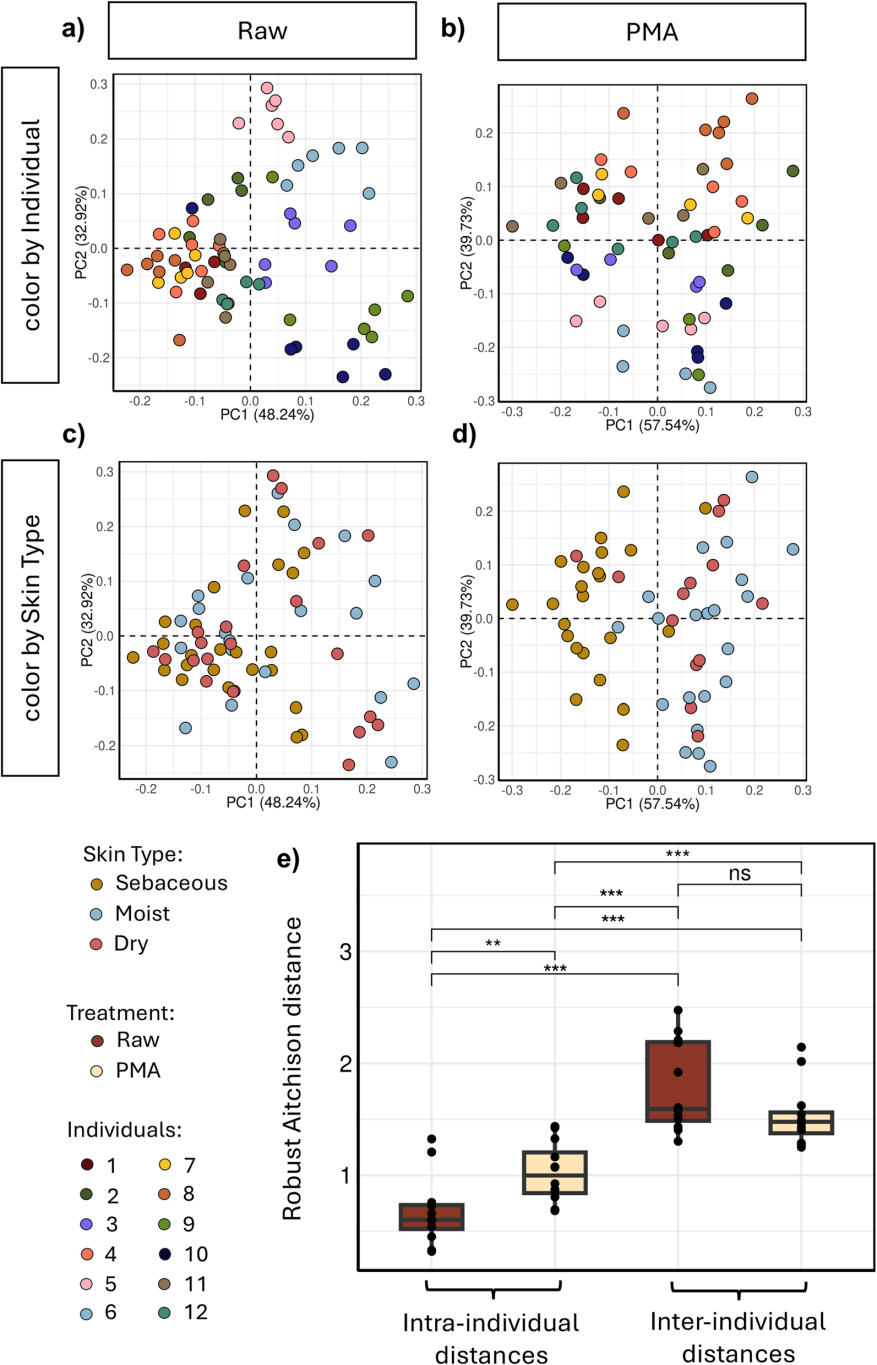
Characterizing the living population of the skin microbiome reveals stronger beta-diversity patterns across individuals. Robust Aitchison principal component analysis (RPCA) plots of samples either **a**, **c** untreated (raw) or **b**, **d** PMA-treated prior to sequencing. Plots are colored either by **a**, **b** individual or by **c**, **d** skin type. **e** Average distances between samples either from the same individuals or between samples from the same skin type but from different individuals (Wilcoxon test for all individuals (multiple test correction “FDR”). ***p < 0.001, **p < 0.01, *p < 0.05, ns: not significant)

Principal coordinate analysis (PCoA) of the robust Aitchison distance matrix (RPCA) calculated on all samples showed that PMA treatment did not affect sample diversity (Fig. S5, Table 1), indicating no artificial bias. RPCA calculated on the raw samples was consistent with data in current literature and showed that clustering was strongest by volunteer compared to any other metadata variable (Fig. 2a, b, Table 1, PERMANOVA) [66, 67]. PMA-treated samples clustered more strongly by skin type than by volunteer, with a significant increase in intraindividual distances (Fig. 2e). The nonsignificant decrease in inter-individual distances in PMA-treated samples compared to raw samples suggests that the living component of skin microbiomes (from the same skin sites) across volunteers is modestly more similar than previously measured. Collectively, these results provide a quantitative measure of relic DNA’s contribution to intraindividual similarity within the skin microbiome, showing that its depletion significantly reduces the microbiome’s apparent personalization (Wilcoxon rank-sum test, FDR-corrected *p* = 0.0035).

To evaluate whether the relic-DNA portion of the samples exhibits personalization, we performed non-metric multidimensional scaling (NMDS) on the robust Aitchison distances computed using only the relic-DNA proportions across all samples with both the raw and corresponding PMA treatment conditions sequenced (*n* = 51 samples) (see the “Materials and methods”). The NMDS results indicated no clustering by individual, with the histogram of distances not following a normal distribution characteristic of sample-type clustering (Fig. S5c, d). Additionally, PERMANOVA results indicated no significant grouping by individual or skin type (*F*-statistic < 2, *p*-value > 0.05). Taken together, the overall results do not support statistically or biologically meaningful grouping of samples by relic DNA alone, and the personalization observed in raw samples is absent in the relic-DNA fraction.

### Total and live cell quantification shows divergent live fraction across the body

Absolute bacterial quantification (bacteria/cm^2^) was measured using flow cytometry on raw (total bacteria) and PMA-treated (live bacteria only) samples (Table S3). Analysis included samples meeting sequencing and flow cytometry gating criteria (*n* = 135: 67 PMA treated, 68 untreated). Method verification across technical replicates from the same volunteer at different time points showed strong correlations for raw (Spearman *r*^2^ = 0.89) and PMA-treated samples (Spearman *r*^2^ = 0.56) (Fig. S7a, d; see the “Materials and methods”).

PMA treatment significantly reduced bacterial counts across all samples (paired Wilcoxon, FDR-corrected *p* < 0.001). Body site grouping also showed significant decreases in bacteria/cm^2^, except for forehead sites, where the live fraction (%) remained high at ~ 88% (Table 2). This elevated viability likely reflects the abundance of nutrient-rich sebum on the forehead (highest density of pilosebaceous units across all body sites [68]), which promotes bacterial proliferation, including *C. acnes* [69]. The larger secretion rates from the abundant pilosebaceous units on the forehead may also contribute to greater live and total cells collected from these sites via swabbing over other sites. Live fraction computed across all individuals showed no significant difference for any given volunteer (Fig. S6a, b), indicating no set of samples affected observed viability across samples (Kruskal–Wallis multiple group comparisons, *F* 16.65, *p* 0.12). The interquartile range (IQR) of estimated bacteria/cm^2^ dropped between raw and PMA-treated samples for all body sites but was only significant for popliteal crease from the moist site (Levene’s test for homogeneity of variance, *F*-stat 4.62, *p* < 0.05), with most body sites still having high variance in measured live cell densities. This suggests that the live cell burden across body sites may be an individual trait.

**Table 2:** Absolute number of live bacteria per square centimeter of the skin. Numbers equal median (IQR (Q3–Q1)) flow cytometry measurements

Body site	Total cells (cells per square centimeter) Median (IQR)	Live cells (cells per square centimeter) Median (IQR)	Live fraction (%) Median (IQR)	*p-value* (Wilcox_paired)
Abdomen (*n* = 11)	5155 (11,528–919)	905 (2695–50)	16.47 (31–5)	*
Antecubital crease (*n* = 12)	10,111 (23,988–3905)	814 (7615–61)	12.6 (35–6)	*
Forearm (*n* = 12)	3729 (5603–1792)	247 (1363–49)	6.31 (33–3)	**
Forehead (*n* = 11)	33,508 (115,790–5019)	29,088 (114,620–4251)	87.72 (115–73)	ns
Popliteal crease (*n* = 11)	2527 (3082–2062)	288 (513–91)	10.23 (19–3)	*
Upper back (*n* = 12)	83,508 (180,906–13,171)	37,047 (87,649–4096)	48.86 (64–32)	**

Paired Wilcoxon test

^***^p < 0.005

^**^p < 0.01

^*^p < 0.05, *ns* non-significant

### Relic-DNA depletion reveals a genuine preferential abundance of bacteria across skin sites

**Fig. 3:**
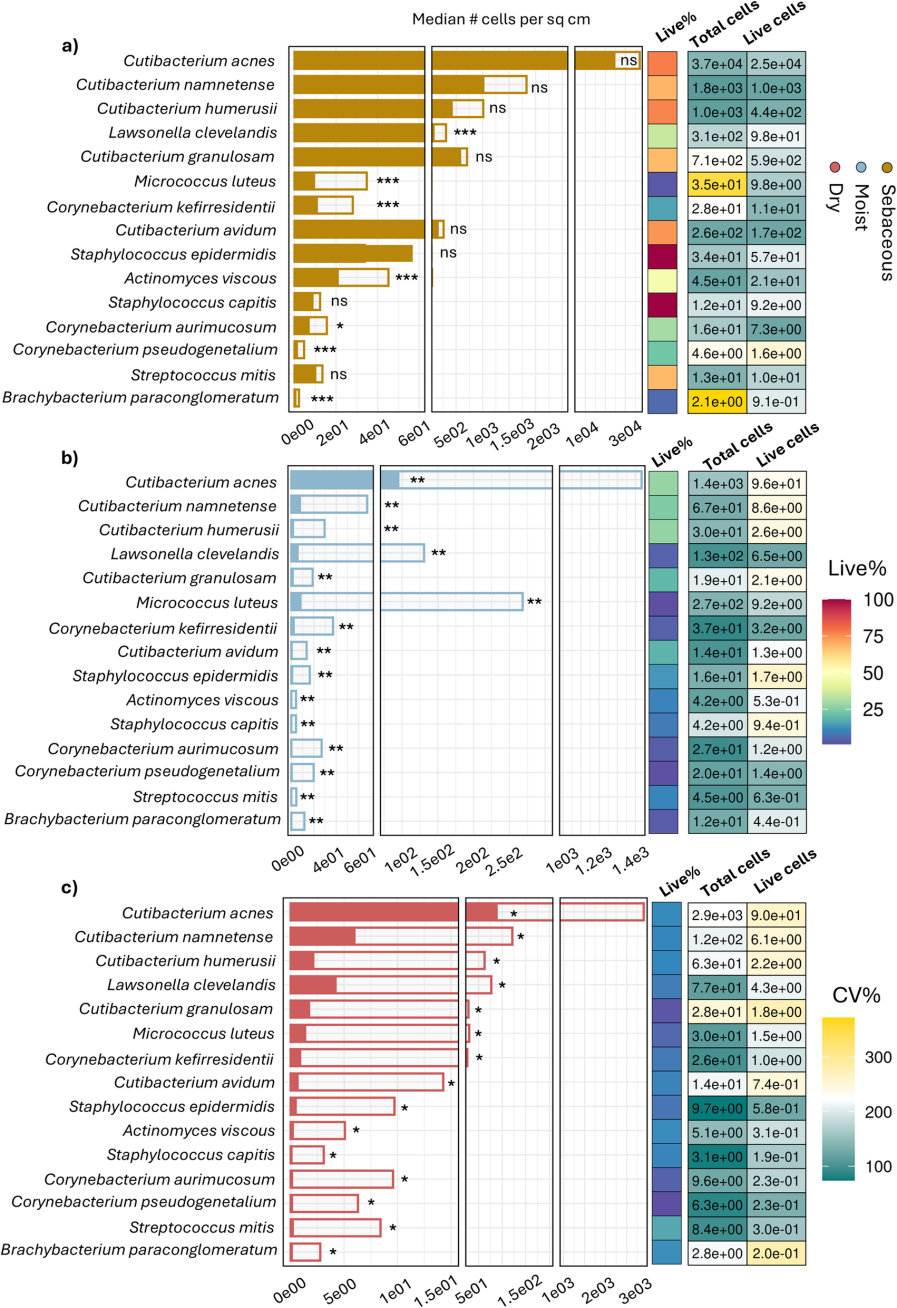
Absolute abundance of taxa across all sites reveals differential live cell levels across all skin types. **a**, **b**, **c** Stacked bars: cells per square centimeter of species identified across all samples at an *RPKM* > 0.05%, arranged in descending order of median relative abundance, in either raw samples (hollow bar) or corresponding PMA-treated samples (overlaid solid bars), grouped by **a** sebaceous sites (*n* = 23), **b** moist sites (*n* = 17), or **c** dry sites (*n* = 11). Paired Wilcoxon test computed by taxa and treatment (raw vs PMA) with multiple testing “FDR” correction: ***p < 0.001, **p < 0.01, *p < 0.05, ns non-significant. Heatmaps: Percentage of live population, and median total cell and live cell counts shaded by coefficient of variance across individuals, for all taxa

**Fig. 4:**
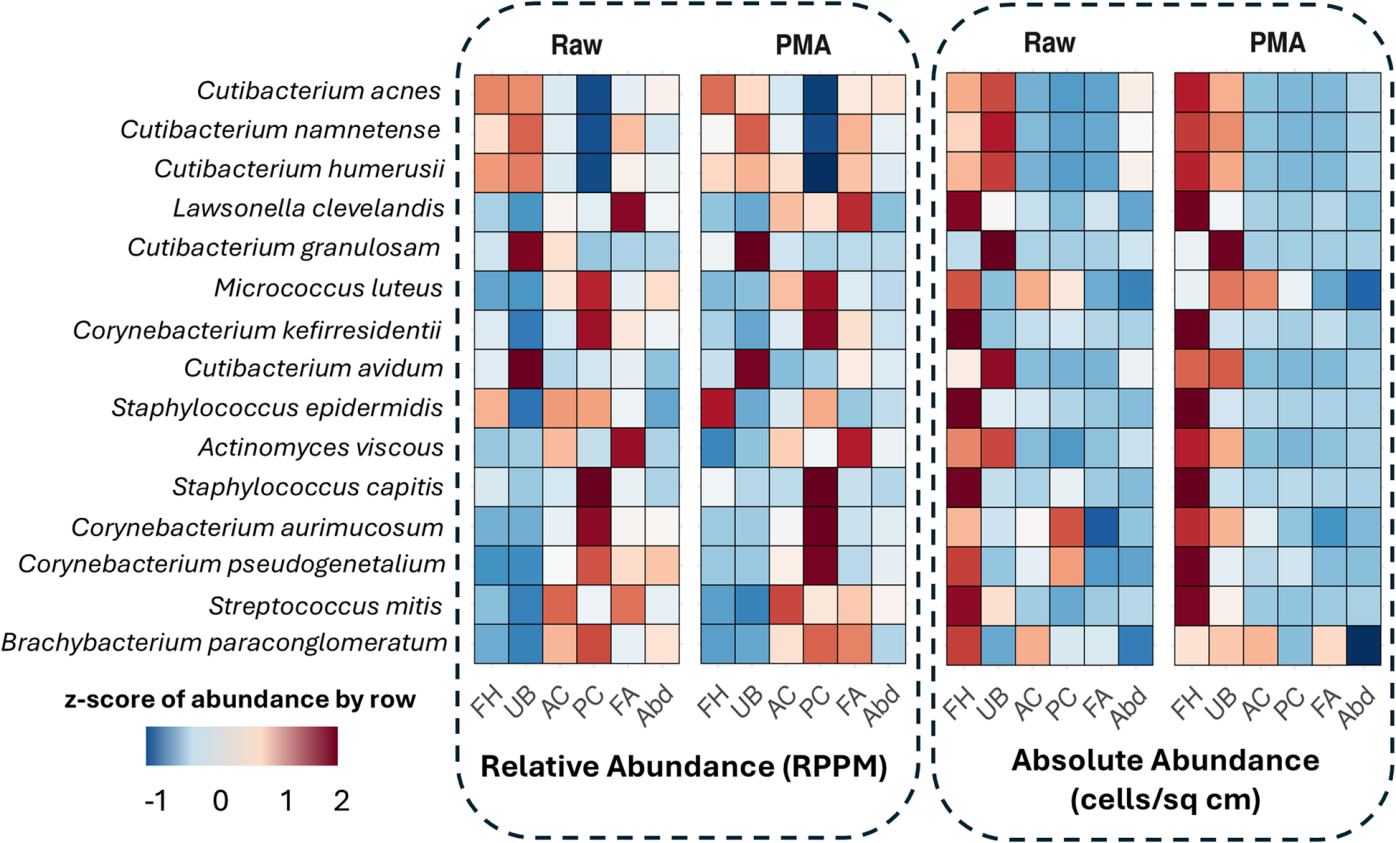
Relic-DNA depletion reveals a genuine preferential abundance of bacteria across skin sites. Heatmap of median (left grid) relative abundance (RPKM) and (right grid) absolute abundance (cells per square centimeter) in raw vs PMA samples for identified species at *RPKM* > 0.05%, arranged in descending order of median relative abundance. Values are z-scored by row in each group

The live fraction (%) of each taxon across different sampling sites was computed by combining results of metagenomics analysis (corrected for read length and genome size) and flow cytometry (see the “Materials and methods”). This analysis was limited to body sites from individuals whose samples met gating criteria and minimum DNA sequencing requirements for both raw and PMA-treated versions (*n* = 102, 51 raw + 51 PMA). Consistent with previous shotgun metagenomic surveys of the skin microbiome [8, 60–62], we found *C. acnes* to be the most abundant taxa across all body sites swabbed for all individuals (Fig. 3a, b, c). However, the live percent of *C. acnes* varied significantly depending on the site swabbed, and we observed that even this most dominant taxa exhibited a reduction of more than 90% in bacterial density when comparing raw and PMA-treated samples in both moist (1658 total bacteria/cm^2^ to 136 live bacteria/cm^2^, median, *n* = 18) and dry (2903 total bacteria/cm^2^ to 90 live bacteria/cm^2^, median, *n* = 11) sites (Table S3). Owing to the high viability of cells from the forehead (Fig. 4a), and the large variance observed across individuals, several *Staphylococcus* taxa, like *Staphylococcus epidermidis* and *S. capitis*, had median live fractions higher than 100%. However, most bacterial species had absolute counts below five detectable bacterial cells/cm^2^ across all skin types from all individuals. These counts were even lower when considering only the live bacterial cells from PMA-treated samples (average paired Wilcoxon *p*-value < 0.001 for all taxa in all samples raw vs PMA).

Next, we evaluated site-specific composition of the skin microbiome, differentiating between total cells and live cells. Sequencing results and corresponding absolute abundance enumerations from raw samples supported earlier findings that report preferential abundance of *Micrococcus*, *Staphylococcus*, and *Corynebacterium* species, such as *Micrococcus luteus*, *S. capitis*, and *Corynebacterium pseudogenetalium*, in moist, humid regions like the antecubital fossa and popliteal crease (Fig. 4a, Table S3) [1, 60, 61]. However, measurements from corresponding PMA-treated samples suggest that this preferential abundance may be influenced by the presence of relic DNA, since live cell counts of *Staphylococcus* and *Corynebacterium* species were always higher in forehead sites, while *M. luteus* had similar live counts between upper back and antecubital crease sites (Fig. 4b). Similarly, measurements of raw samples bacteria density from dry sites confirmed previously reported preferential abundance of *Streptococcus mitis* at these sites [1, 60]; however, corresponding PMA-treated swabs demonstrated a clear dominance of live *S. mitis* cells in sebaceous sites compared to all other regions (Fig. 4a, b). A complete record of the absolute abundance, live%, and standard deviation of the common skin microbiome taxa identified in this study is provided in Table S4. These findings suggest there may be variable detection of certain species at different sites across individuals due to host-specific behavior and activity, but the levels of live bacteria are mainly consistent with their survival biased toward the more lipid-rich sebaceous sites.

## Discussion

We characterized and quantified the absolute abundance of the live skin microbiome by combining relic-DNA depletion, load determination using flow cytometry, and metagenomic sequencing. Using PMA treatment and parallel processing with and without PMA, we assessed the impact of relic DNA on the characterization and quantification of the microbiome in low-biomass samples using skin swabs. We measured live bacterial cells across 6 skin sites spanning 3 different skin types, i.e., sebaceous, moist, and dry, from 12 individuals.

In sebaceous regions like the forehead and upper back, we counted a median (interquartile range Q3–Q1) cell density of 2.91e + 04 (11.5e + 04–0.4e + 04) and 3.70e + 04 (8.76e + 04–0.41e + 04) live bacteria/cm^2^, respectively, aligning with previous cultivation-based estimates that described 1e + 04–1e + 05 CFU/cm^2^ [28, 70]. Moist sites like antecubital and popliteal crease harbored 8.14e + 02 (7.62e + 03–0.6e + 02) and 2.88e + 02 (5.13e + 02–0.9e + 02) live bacteria/cm^2^, respectively. Note that this is an order of magnitude lower than prior estimates of 1e + 04 CFU/cm^2^ [28]; however, this discrepancy is likely due to the inclusion of axilla samples in these studies, which can contain as many as 1e + 06 CFU/cm^2^ [30]. Dry sites like forearm and abdomen contained only 2.5e + 02 (1.36e + 03–0.5e + 02) and 9.0e + 02 (2.69e + 03–0.5e + 02) live bacteria/cm^2^, respectively, similar to the lower range of these prior culture-based estimates [7, 29, 30]. To avoid exposing intact cells to lytic substances, swabbing for this study was performed without detergents unlike other swabbing studies, and the surface areas of the sampled body sites were expanded (Table S2) to ensure sufficient microbial cell collection. This difference in sampling methodology could also account for the variations between our calculated abundances and some levels reported in previous studies. This variability can also be attributed to the larger sampling area and the inherent inconsistencies between studies in the swabbing method, such as the pressure applied during swabbing or the number of swab rotations performed. To minimize these variations in our study, the same individual swabbed all volunteers, and all the swabbing was completed over three consecutive days (see the “Materials and methods” for more details). Further robustness can be achieved by incorporating rigorous negative controls and blanks with sequencing, along with repeated collections from volunteers to normalize variations arising from sampling [9].

Metagenomic analysis of raw and PMA-treated samples recapitulates prior reports that skin microbiomes are specific to individuals [61, 67]. Our approach with PMA-treated samples also showed clustering by individuals; however, PERMANOVA measured this clustering to be much lower than by skin type, with intraindividual samples significantly increasing for PMA-treated samples. Analyzing the relic-DNA portion only in samples found no clustering by individuals (Fig. S3c), suggesting that personalization of the microbiome is still a trait of the live skin microbiome, albeit not as significant as seen from samples biased with relic DNA. However, a significant portion of personalization in the microbiome can come from microbial dark matter, which could not be resolved from either skin-specific [8] or general databases [54] used in this study [71]. Relic DNA has been shown to serve as a nutrient source for microbes in deep-sea marine systems [72], as well as promoting biofilm formation in clinical isolates of common skin taxa like *Staphylococcus aureus* taken from paranasal sinuses of patients with chronic rhinosinusitis [73]. Thus, the nature of relic DNA and its difference between individuals may impact the overall diversity of the live microbiome, an aspect that could be evaluated in future studies.

The compositional analysis of PMA-treated and untreated samples revealed modestly significant differences based on the PMA-index metric only for a few, low abundant taxa, only at certain body sites (Fig. S3), suggesting that relic DNA on the skin surface does not substantially under- or overrepresent most taxa or groups of species. In contrast, prior studies using amplicon sequencing have reported large PMA-index values with significant compositional differences between raw and PMA-treated samples [26]. This highlights a key distinction between shotgun sequencing and amplicon-based approaches, which has been well characterized for microbiome studies [45, 46, 74]. While both methods consistently identify the top taxonomic members, shotgun sequencing captures significantly greater diversity within samples compared to amplicon-based sequencing [46, 75]. However, the technical differences in the two methods, such as size of reads sequenced (~ 75–90 bp vs ~ 400 bp) and sequencing platforms used and sequencing strategy (2 × 150 bp vs 2 × 300 bp), limit a direct comparison between either approach.

PMA treatment can introduce biases toward certain bacterial species due to differences in cell wall properties and biochemistry [76]. However, since skin swab samples are low in diversity and biomass, this bias should be minimal, as demonstrated by studies using mock multi-member or co-culture communities [77] or other low abundant sample studies [78]. Furthermore, concordance between PMA flow cytometry and CFU plating for overnight cultures of the skin bacteria *S. epidermidis* and *Escherichia coli* (Fig. S9), as well as that seen for other skin species in other studies [26, 79], suggests that PMA treatment does not significantly bias the quantification of live cells for these species. That said, crucial differences exist between lab-cultivated, in vitro, and in situ conditions versus the natural skin surface environment, which promotes biofilm formation and enhanced aggregation. To this end, we also compared concordance between flow cytometry estimates between samples collected from the same individual at different time points (designated as volunteer 1 and volunteer 12 throughout the study). A positive correlation between the raw samples (Spearman, *R* = 0.83, *p* 0.058) and the PMA samples (Spearman, *R* = 0.9, *p* 0.083) suggests heterogeneities of PMA interactions with the microbes due to cell wall biochemistries are minimal. The forehead sample lies just outside of the 95% confidence interval, but this could be a sign of the high temporal instability of the forehead microbiome [60]. We also measured high correlation between the relative (Spearman R for raw: 0.97, *p* < 0.001; *PMA*: 0.96, *p* < 0.001) and absolute (Spearman R for raw: 0.86, *p* < 0.001; *PMA*: 0.73, *p* < 0.001) abundance of taxa obtained from the volunteer on the two separate sampling times (Fig. S7b and c, e and f). Taken together, PMA bias and/or inefficiencies could be present in the skin swab samples despite not showing up in cultivated and/ or mock community controls, but these biases are likely minimal due to the low diversity and low biomass nature of skin swabs.

We also estimated cell counts for some of the commonly found skin taxa from both raw and the corresponding PMA-treated samples. Our measurements confirmed *C. acnes* to be the most abundant taxa across all the six different body sites measured, with the highest live-cell burden of 2.5e + 04 (1.01e + 04–0.37e + 04) live cells per cm^2^ measured from the forehead. Given that there are roughly 400–900 sebaceous pores per cm^2^ on the forehead for the general adult population [80], our results suggest that there are at least 28 live cells of *C. acnes* on average per sebaceous pore, which aligns with recent findings that pores are colonized by unique *C. acnes* lineages [81].

Precise quantification of the total number of live cells is crucial for our understanding of the skin microbiome’s impact on health and disease and for developing effective treatments. Disease severity has often been correlated with pathogen density [32, 82]. However, there have been several reports where microbiome data and disease progression seem to be contradictory. For example, studies on atopic dermatitis (AD) have reported non-changing levels of commensals in AD samples pre- and post-flares [83]. Others have reported no difference in diversity between healthy sites and sites with seborrheic dermatitis (SD) despite differences in live cell counts [84]. Raw and PMA-treated swabs collected from the flexure regions of the inner elbow and behind the knee (moist) and forearm (dry) in our study showed no significant differential abundance of the top bacterial taxa at those sites (Fig. S3) nor a significant drop in diversity (Fig. S4). However, quantification of the absolute abundances revealed significantly lower live cell densities, especially at moist and dry sites, with less than 10% of the total cells alive for most taxa (Table S3). This low percentage of live bacterial cells, i.e., commensals, may explain why flexure sites, hands, and general extremities (dry) are more prone to infection by pathogens competing for the same niche as commensals [85, 86], a fact that will be explored in future studies.

Skin microbiome studies incorporating relic-DNA depletion can successfully discriminate between dead and live cells, elucidating disease progression in a quantitative way. Furthermore, the approach could guide the design of therapies focused on supporting the live bacterial cell population. With dry sites like the forearm being the lowest in bacterial cell viability with a live fraction of just 5%, our study may provide a plausible explanation for why certain skin sites have such a high susceptibility to infection, which would be crucial for guiding the design of skin microbiome therapeutics founded on a better understanding of host-microbe interactions.

## Supplementary Information

Supplementary Material 1: Figure S1. Mapping parameters and relative abundance of all samples sequenced part of the study. a) Box plot of percentage of reads mapped to the reference human genome (GRCh38.p14) after quality and swab + blank control filtering. b) Bar plot of average total reads per sample that passed quality filtering for swab/blank filtering, then to host genome filtering, and then for mapping to the database and the reads that mapped (bacterial genomes only in SMGC). c) Box plot of percentage of reads mapped to the reference database (SMGC) after quality, swab and blank and human filtering. d) Density plot of the ratio of the reference genome in SMGC database covered in all samples in the dataset, which was used to constrain alignment and read assignment using Zebra. Figure S2: Principal component analysis of Raw and PMA-treated samples. a) PCA of Raw samples. b) PCA of PMA-treated samples. c) Relative abundance bar plots of identified taxa across all samples sequenced as part of the study. Identified species with median RPKM > 0.05% reported. Figure S3: PMA index and alpha diversity index shows no significant change between Raw and PMA-treated samples. a) Heatmap of the PMA_index (formula above) computed on top taxa identified across all samples averaged by body site (FH: forehead, UB: upper back, AC: antecubital crease, PC: popliteal crease, FA: forearm, Abd: abdomen). Heatmaps are shaded by the PMA-index score, where a score greater than 0.5 indicates an underestimation of that taxa in samples, and lesser than 0.5 suggests an overestimation of that taxa in samples. Inset values represent the standard deviation in the computed PMA_index across individuals and the paired Wilcoxon test with multiple testing“fdr” corrections computed for each taxa by body site : *** *p*<0.005, ** *p*<0.01, * *p<*0.05, ns non-significant. Body Site acronyms: FH (forehead), UB (upper back), AC (antecubital crease), PC (popliteal crease), FA (forearm), Abd (abdomen). Figure S4: Alpha diversity of samples reveals no significant bias from relic-DNA in skin microbiome samples. a) Correlation plots between sequencing depth of all samples (library size) and a) Shannon diversity or b) Simpson evenness alpha diversity metrics. Spearman correlation of -0.323 for both a) and b), suggesting no effect from sequencing depth on the computed alpha diversity. c) Box plot of Shannon diversity of all samples in Raw vs PMA-treated samples grouped by body site and colored by skin type. Both Raw and PMA-treated samples retain similar patterns for cross skin-type diversities. (Kruskal–Wallis multi-group non-parametric test computed on Skin Type, *n* = 57 (PMA) and *n* = 66 (Raw)). d) Box plot of Shannon diversity comparisons between samples from the same body site between raw and PMA treatments shows no significant difference (Wilcoxon rank-sum test, multiple comparisons “fdr” correction). e) Box plot of Simpson evenness of all samples in Raw vs PMA-treated samples grouped by body site and colored by skin type. Both Raw and PMA-treated samples retain similar patterns for cross skin-type diversities. (Kruskal–Wallis multi-group non-parametric test computed on Skin Type, *n* = 57 (PMA) and *n* = 66 (Raw)). f) Box plot of Simpson evenness comparisons between samples from the same body site between raw and PMA treatments shows no significant difference (Wilcoxon rank-sum test, multiple comparisons “fdr” correction). Figure S5: relic-DNA in samples is not personalized. a) PCoA (Principal Coordinate Analysis) on the robust aitchison distances between all samples (*n* = 123) in the dataset, colored by treatment. b) Histogram plot of the robust aitchison distances between all samples (raw + PMA). c) NMDS plot of the robust aitchison distances between the relative abundance of the relic-DNA proportion only in all samples aligned to Web Of Life database (Zhu et al. 2019) colored by volunteer. d) Histogram plot of the robust aitchison distances between samples calculated using only the relic-DNA portion of the samples. e) PERMANOVA results computed on robust aitchison distances between the relic-DNA proportion in samples only to test grouping by Volunteer, Body Site or Skin Type. (*p *values >0.05, ns (not significant)). Figure S6: Comparison of total cells vs live cells and percentage live across all volunteers. a) Box plot of cell per sq cm enumerated from all body sites in an volunteer indicates no volunteer had a significant drop in cell counts between treatments. (Wilcoxon paired test, FDR multiple test corrections. *P > *0.05 ns) b) Live fraction (%) computed across all samples from an volunteer (Kruskal-Wallis multiple group comparisons test and Dunn’s pairwise comparison). Fig S7: Correlation plots between Volunteer 1 time-point 1 and time-point 2 (Volunteer 12 in sample sheet) sampling. a)-c) Correlation plots with 95% confidence intervals for all matching raw samples from Volunteer 1 and Volunteer 12 based on a) flow cytometry, b) relative abundance (rpkm) and c) absolute abundance of taxa. d) – f) Correlation plots with 95% confidence intervals for all matching PMA_treated samples from Volunteer 1 and Volunteer 12 based on d) flow cytometry, e) relative abundance (rpkm) and f) absolute abundance of taxa. Figure S8: Representative example of the gating strategy applied to all flow cytometry samples. Flow cytometry gating strategy adapted from Marotz et al. and Props et al. Skin swab samples collected in 1X PBS were vortexed at maximum speed. 400 uL of this was filtered across a 5-μm filter to remove human cells and swab lint, stained with SYBR green (0.1X final), and processed on a Sony SH800 instrument with Spherotech counting beads. The threshold was set on the FL1 detector. a) The first gate selects for events with enhanced 525-nm specific emission to select DNA-positive events. b) Doublets were excluded by selecting only events following a linear trend between FL1 height and area. c) Human cells are excluded by their large size on forward (FSC-A) and side (SSC-A) scatter area. d) Representative example of cell counts obtained from Raw (blue) vs PMA-treatd (red) samples across the body sites sampled using the gating strategy. Figure S9: Correlation between an overnight control of (a) *Escherichia coli *and (b) *S. epidermidis *CFU counts and counts estimated from flow cytometry of PMA-treated aliquots of the cultures. Supplementary Table S1: Metadata of individuals involved in the study. Supplementary Table S2: Body site metadata and dimensions of plastic patterns.

Supplementary Material 2: Table S3. Median (IQR) absolute abundance of total and live cells of all taxa across all skin types and body sites.

Supplementary Material 3: Table S4. Alignment statistics and mapping rates for all samples.

## Data Availability

The metagenomic sequencing data generated in this study have been deposited in the NCBI Sequence Read Archive database under accession code PRJNA1118035 (https://www.ncbi.nlm.nih.gov/sra/?term=PRJNA1118035). The sequencing data and the associated metadata can also be viewed together for the convenience of the reviewers via the SRA Run Selector site using this public link (https://trace.ncbi.nlm.nih.gov/Traces/study/?acc=PRJNA1118035&o=acc_s%3Aa). The codes used to preprocess the metagenomic data and analyze the results can be publicly accessed at Zenodo and at https://github.com/dthirupp/DeadOrAlive-SkinMicrobiome. (10.5281/zenodo.12727359). The reviewers may also access the same code via this github link if preferred (https://github.com/ZenglerLab/DeadOrAlive-SkinMicrobiome).

## Abbreviations

PMA: Propidium monoazide
PCR: Polymerase chain reaction
RPCA: Robust Aitchison principal component analysis
PERMANOVA: Permutational multivariate analysis of variance
FDR: False discovery rate
AD: Atopic dermatitis
SD: Seborrheic dermatitis
IGM: Institute of Genomic Medicine
IRB: Institutional Review Board
FSC: Forward scatter
BSC: Backward scatter
FL: Fluorescence filters
HS: High sensitivity
CPM: Counts per million
OGU: Operational genomic units

## References

[1] Byrd AL, Belkaid Y, Segre JA. The human skin microbiome. Nat Rev Microbiol. 2018;16:143–55. 29332945 10.1038/nrmicro.2017.157

[2] Segre JA. Epidermal barrier formation and recovery in skin disorders. J Clin Invest. 2006;116:1150–8. 16670755 10.1172/JCI28521 PMC1451215

[3] Gallo RL. Human skin is the largest epithelial surface for interaction with microbes. J Investig Dermatol. 2017;137:1213–4. 28395897 10.1016/j.jid.2016.11.045 PMC5814118

[4] Grice EA, Kong HH, Conlan S, Deming CB, Davis J, Young AC, et al. Topographical and temporal diversity of the human skin microbiome. Science. 2009;324:1190–2. 19478181 10.1126/science.1171700 PMC2805064

[5] Findley K, Oh J, Yang J, Conlan S, Deming C, Meyer JA, et al. Topographic diversity of fungal and bacterial communities in human skin. Nature. 2013;498:367–70. 23698366 10.1038/nature12171 PMC3711185

[6] Timm CM, Loomis K, Stone W, Mehoke T, Brensinger B, Pellicore M, et al. Isolation and characterization of diverse microbial representatives from the human skin microbiome. Microbiome. 2020;8:58. 32321582 10.1186/s40168-020-00831-y PMC7178971

[7] Evans CA, Smith WM, Johnston EA, Giblett ER. Bacterial flora of the normal human skin*. J Investig Dermatol. 1950;15:305–24. 14779047 10.1038/jid.1950.105

[8] Saheb Kashaf S, Proctor DM, Deming C, Saary P, Hölzer M, NISC Comparative Sequencing Program, et al. Integrating cultivation and metagenomics for a multi-kingdom view of skin microbiome diversity and functions. Nat Microbiol. 2022;7:169–79. 34952941 10.1038/s41564-021-01011-w PMC8732310

[9] Kong HH, Andersson B, Clavel T, Common JE, Jackson SA, Olson ND, et al. Performing skin microbiome research: a method to the madness. J Invest Dermatol. 2017;137:561–8. 28063650 10.1016/j.jid.2016.10.033 PMC5468751

[10] The Human Microbiome Project Consortium, Huttenhower C, Gevers D, Knight R, Abubucker S, Badger JH, et al. Structure, function and diversity of the healthy human microbiome. Nature. 2012;486:207–14. 22699609 10.1038/nature11234 PMC3564958

[11] Costello EK, Lauber CL, Hamady M, Fierer N, Gordon JI, Knight R. Bacterial community variation in human body habitats across space and time. Science. 2009;326:1694–7. 19892944 10.1126/science.1177486 PMC3602444

[12] Saheb Kashaf S, Kong HH. Adding fuel to the fire? The skin microbiome in atopic dermatitis. J Investig Dermatol. 2024;144:969–77. 38530677 10.1016/j.jid.2024.01.011 PMC11034722

[13] Props R, Monsieurs P, Mysara M, Clement L, Boon N. Measuring the biodiversity of microbial communities by flow cytometry. Methods Ecol Evol. 2016;7:1376–85.

[14] Walters W, Hyde ER, Berg-Lyons D, Ackermann G, Humphrey G, Parada A, et al. Improved bacterial 16S rRNA gene (V4 and V4-5) and fungal internal transcribed spacer marker gene primers for microbial community surveys. mSystems. 2015;1:00009–15. 10.1128/mSystems.00009-15 PMC5069754 27822518

[15] Chen Y, Knight R, Gallo RL. Evolving approaches to profiling the microbiome in skin disease. Front Immunol. 2023;14:1151527. 37081873 10.3389/fimmu.2023.1151527 PMC10110978

[16] Bjerre RD, Hugerth LW, Boulund F, Seifert M, Johansen JD, Engstrand L. Effects of sampling strategy and DNA extraction on human skin microbiome investigations. Sci Rep. 2019;9:17287. 31754146 10.1038/s41598-019-53599-z PMC6872721

[17] Bairoliya S, Koh Zhi Xiang J, Cao B. Extracellular DNA in environmental samples: occurrence, extraction, quantification, and impact on microbial biodiversity assessment. Appl Environ Microbiol. 2022;88:e01845-21. 34818108 10.1128/aem.01845-21 PMC8824265

[18] Rogers GB, Marsh P, Stressmann AF, Allen CE, Daniels TVW, Carroll MP, et al. The exclusion of dead bacterial cells is essential for accurate molecular analysis of clinical samples. Clin Microbiol Infect. 2010;16:1656–8. 20148918 10.1111/j.1469-0691.2010.03189.x

[19] Yap M, O’Sullivan O, O’Toole PW, Cotter PD. Development of sequencing-based methodologies to distinguish viable from non-viable cells in a bovine milk matrix: a pilot study. Front Microbiol. 2022;13:1036643. 36466696 10.3389/fmicb.2022.1036643 PMC9713316

[20] Kumar MS, Slud EV, Okrah K, Hicks SC, Hannenhalli S, Corrada BH. Analysis and correction of compositional bias in sparse sequencing count data. BMC Genomics. 2018;19:799. 30400812 10.1186/s12864-018-5160-5 PMC6219007

[21] Gloor GB, Macklaim JM, Pawlowsky-Glahn V, Egozcue JJ. Microbiome datasets are compositional: and this is not optional. Front Microbiol. 2017;8:2224. 29187837 10.3389/fmicb.2017.02224 PMC5695134

[22] Zaramela LS, Tjuanta M, Moyne O, Neal M, Zengler K. synDNA—a synthetic DNA spike-in method for absolute quantification of shotgun metagenomic sequencing. mSystems. 2022;7:e00447-22. 36317886 10.1128/msystems.00447-22 PMC9765022

[23] Stämmler F, Gläsner J, Hiergeist A, Holler E, Weber D, Oefner PJ, et al. Adjusting microbiome profiles for differences in microbial load by spike-in bacteria. Microbiome. 2016;4:28. 27329048 10.1186/s40168-016-0175-0 PMC4915049

[24] Morton JT, Marotz C, Washburne A, Silverman J, Zaramela LS, Edlund A, et al. Establishing microbial composition measurement standards with reference frames. Nat Commun. 2019;10:2719. 31222023 10.1038/s41467-019-10656-5 PMC6586903

[25] Lennon JT, Muscarella ME, Placella SA, Lehmkuhl BK. How, when, and where relic-DNA affects microbial diversity. Zhou J, editor. mBio. 2018;9:e00637-18. 29921664 10.1128/mBio.00637-18 PMC6016248

[26] Acosta EM, Little KA, Bratton BP, Lopez JG, Mao X, Payne A, et al. Bacterial DNA on the skin surface overrepresents the viable skin microbiome. eLife. 2023;12. Available from: https://elifesciences.org/reviewed-preprints/87192. Cited 2024 Jul 8. 10.7554/eLife.87192 PMC10328497 37389570

[27] Gao Z, Perez-Perez GI, Chen Y, Blaser MJ. Quantitation of major human cutaneous bacterial and fungal populations. J Clin Microbiol. 2010;48:3575–81. 20702672 10.1128/JCM.00597-10 PMC2953113

[28] Grice EA, Kong HH, Renaud G, Young AC, Bouffard GG, Blakesley RW, et al. A diversity profile of the human skin microbiota. Genome Res. 2008;18:1043–50. 18502944 10.1101/gr.075549.107 PMC2493393

[29] Baviera G, Leoni MC, Capra L, Cipriani F, Longo G, Maiello N, et al. Microbiota in healthy skin and in atopic eczema. Biomed Res Int. 2014;2014:436921. 25126558 10.1155/2014/436921 PMC4122000

[30] Leyden JJ, McGinley KJ, Nordstrom KM, Webster GF. Skin microflora. J Investig Dermatol. 1987;88:65–72. 10.1111/1523-1747.ep12468965 3102625

[31] Zengler K, Toledo G, Rappé M, Elkins J, Mathur EJ, Short JM, et al. Cultivating the uncultured. Proc Natl Acad Sci. 2002;99:15681–6. 12438682 10.1073/pnas.252630999 PMC137776

[32] Nakatsuji T, Chen TH, Narala S, Chun KA, Two AM, Yun T, et al. Antimicrobials from human skin commensal bacteria protect against staphylococcus aureus and are deficient in atopic dermatitis. Sci Transl Med. 2017;9:eaah468. 10.1126/scitranslmed.aah4680 PMC5600545 28228596

[33] Johnson JS, Spakowicz DJ, Hong B-Y, Petersen LM, Demkowicz P, Chen L, et al. Evaluation of 16S rRNA gene sequencing for species and strain-level microbiome analysis. Nat Commun. 2019;10:5029. 31695033 10.1038/s41467-019-13036-1 PMC6834636

[34] Fittipaldi M, Nocker A, Codony F. Progress in understanding preferential detection of live cells using viability dyes in combination with DNA amplification. J Microbiol Methods. 2012;91:276–89. 22940102 10.1016/j.mimet.2012.08.007

[35] Soejima T, Iida K, Qin T, Taniai H, Seki M, Takade A, et al. Photoactivated ethidium monoazide directly cleaves bacterial DNA and is applied to PCR for discrimination of live and dead bacteria. Microbiol Immunol. 2007;51:763–75. 17704639 10.1111/j.1348-0421.2007.tb03966.x

[36] Labaer J, Murugan V, Vorachitti M, Pannala R, Faigel D. Methods for detection and quantification of infectious carbapenem resistant enterobacteriaceae (CRE). 2023. Available from: https://patents.google.com/patent/US11840723B2/en?oq=us11840723. Cited 2024 Dec 30.

[37] Stinson LF, Keelan JA, Payne MS. Characterization of the bacterial microbiome in first-pass meconium using propidium monoazide (PMA) to exclude nonviable bacterial DNA. Lett Appl Microbiol. 2019;68:378–85. 30674082 10.1111/lam.13119

[38] Galazzo G, van Best N, Benedikter BJ, Janssen K, Bervoets L, Driessen C, et al. How to count our microbes? The effect of different quantitative microbiome profiling approaches. Front Cell Infect Microbiol. 2020;10. Available from: https://www.frontiersin.org/journals/cellular-and-infection-microbiology/articles/10.3389/fcimb.2020.00403/full. Cited 2024 Dec 30. 10.3389/fcimb.2020.00403 PMC7426659 32850498

[39] Chu ND, Smith MB, Perrotta AR, Kassam Z, Alm EJ. Profiling living bacteria informs preparation of fecal microbiota transplantations. PLoS ONE. 2017;12:e0170922. 28125667 10.1371/journal.pone.0170922 PMC5268452

[40] Thomas MC, Waugh G, Damjanovic K, Vanwonterghem I, Webster NS, Negri AP, et al. Development of a quantitative PMA-16S rRNA gene sequencing workflow for absolute abundance measurements of seawater microbial communities. Research Square; 2024. 10.1186/s40793-025-00741-2 PMC12220233 40597447

[41] Ren Q, Wei F, Yuan C, Zhu C, Zhang Q, Quan J, et al. The effects of removing dead bacteria by propidium monoazide on the profile of salivary microbiome. BMC Oral Health. 2021;21:460. 34551743 10.1186/s12903-021-01832-5 PMC8456568

[42] Marotz CA, Sanders JG, Zuniga C, Zaramela LS, Knight R, Zengler K. Improving saliva shotgun metagenomics by chemical host DNA depletion. Microbiome. 2018;6:42. 29482639 10.1186/s40168-018-0426-3 PMC5827986

[43] Nguyen LDN, Deschaght P, Merlin S, Loywick A, Audebert C, Daele SV, et al. Effects of propidium monoazide (PMA) treatment on mycobiome and bacteriome analysis of cystic fibrosis airways during exacerbation. PLoS ONE. 2016;11:e0168860. 28030619 10.1371/journal.pone.0168860 PMC5193350

[44] Marotz C, Morton JT, Navarro P, Coker J, Belda-Ferre P, Knight R, et al. Quantifying live microbial load in human saliva samples over time reveals stable composition and dynamic load. mSystems. 2021;6:e01182-20. 33594005 10.1128/mSystems.01182-20 PMC8561659

[45] Lamoureux C, Surgers L, Fihman V, Gricourt G, Demontant V, Trawinski E, et al. Prospective comparison between shotgun metagenomics and Sanger sequencing of the 16S rRNA gene for the etiological diagnosis of infections. Front Microbiol. 2022;13:761873. 35464955 10.3389/fmicb.2022.761873 PMC9020828

[46] Brumfield KD, Huq A, Colwell RR, Olds JL, Leddy MB. Microbial resolution of whole genome shotgun and 16S amplicon metagenomic sequencing using publicly available NEON data. PLoS ONE. 2020;15:e0228899. 32053657 10.1371/journal.pone.0228899 PMC7018008

[47] Meisel JS, Hannigan GD, Tyldsley AS, SanMiguel AJ, Hodkinson BP, Zheng Q, et al. Skin microbiome surveys are strongly influenced by experimental design. J Investig Dermatol. 2016;136:947–56. 26829039 10.1016/j.jid.2016.01.016 PMC4842136

[48] Prest EI, Hammes F, Kötzsch S, van Loosdrecht MCM, Vrouwenvelder JS. Monitoring microbiological changes in drinking water systems using a fast and reproducible flow cytometric method. Water Res. 2013;47:7131–42. 24183559 10.1016/j.watres.2013.07.051

[49] Martin M. Cutadapt removes adapter sequences from high-throughput sequencing reads. EMBnet J. 2011;17:10–2.

[50] Babraham Bioinformatics - FastQC a quality control tool for high throughput sequence data. Available from: https://www.bioinformatics.babraham.ac.uk/projects/fastqc/. Cited 2024 Jul 8.

[51] Langmead B, Salzberg SL. Fast gapped-read alignment with Bowtie 2. Nat Methods. 2012;9:357–9. 22388286 10.1038/nmeth.1923 PMC3322381

[52] Hakim D, Wandro S, Zengler K, Zaramela LS, Nowinski B, Swafford A, et al. Zebra: static and dynamic genome cover thresholds with overlapping references. mSystems. 2022;7:e0075822. 36073806 10.1128/msystems.00758-22 PMC9600373

[53] Zhu Q, Huang S, Gonzalez A, McGrath I, McDonald D, Haiminen N, et al. Phylogeny-aware analysis of metagenome community ecology based on matched reference genomes while bypassing taxonomy. mSystems. 2022;7:e00167-22. 35369727 10.1128/msystems.00167-22 PMC9040630

[54] Zhu Q, Mai U, Pfeiffer W, Janssen S, Asnicar F, Sanders JG, et al. Phylogenomics of 10,575 genomes reveals evolutionary proximity between domains bacteria and archaea. Nat Commun. 2019;10:5477. 31792218 10.1038/s41467-019-13443-4 PMC6889312

[55] Benjamini Y, Hochberg Y. Controlling the false discovery rate: a practical and powerful approach to multiple testing. J Roy Stat Soc: Ser B (Methodol). 1995;57:289–300.

[56] McMurdie PJ, Holmes S. phyloseq: an R package for reproducible interactive analysis and graphics of microbiome census data. PLoS ONE. 2013;8:e61217. 23630581 10.1371/journal.pone.0061217 PMC3632530

[57] Oksanen J, Simpson GL, Blanchet FG, Kindt R, Legendre P, Minchin PR, et al. vegan: Community Ecology Package. 2001. p. 2.6–6.1. Available from: https://CRAN.R-project.org/package=vegan. Cited 2024 Jul 10.

[58] Martino C, Morton JT, Marotz CA, Thompson LR, Tripathi A, Knight R, et al. A novel sparse compositional technique reveals microbial perturbations. mSystems. 2019;4:e00016-19. 30801021 10.1128/mSystems.00016-19 PMC6372836

[59] Bolyen E, Rideout JR, Dillon MR, Bokulich NA, Abnet CC, Al-Ghalith GA, et al. Reproducible, interactive, scalable and extensible microbiome data science using QIIME 2. Nat Biotechnol. 2019;37:852–7. 31341288 10.1038/s41587-019-0209-9 PMC7015180

[60] Oh J, Byrd AL, Park M, Kong HH, Segre JA. Temporal stability of the human skin microbiome. Cell. 2016;165:854–66. 27153496 10.1016/j.cell.2016.04.008 PMC4860256

[61] Oh J, Byrd AL, Deming C, Conlan S, Kong HH, Segre JA. Biogeography and individuality shape function in the human skin metagenome. Nature. 2014;514:59–64. 25279917 10.1038/nature13786 PMC4185404

[62] Smythe P, Wilkinson HN. The skin microbiome: current landscape and future opportunities. Int J Mol Sci. 2023;24:3950. 36835363 10.3390/ijms24043950 PMC9963692

[63] Grice EA, Segre JA. The skin microbiome. Nat Rev Microbiol. 2011;9:244–53. 21407241 10.1038/nrmicro2537 PMC3535073

[64] Lin H, Peddada SD. Analysis of microbial compositions: a review of normalization and differential abundance analysis. NPJ Biofilms Microbiomes. 2020;6:60. 33268781 10.1038/s41522-020-00160-w PMC7710733

[65] Quinn TP, Erb I, Richardson MF, Crowley TM. Understanding sequencing data as compositions: an outlook and review. Bioinformatics. 2018;34:2870–8. 29608657 10.1093/bioinformatics/bty175 PMC6084572

[66] Flores GE, Caporaso JG, Henley JB, Rideout JR, Domogala D, Chase J, et al. Temporal variability is a personalized feature of the human microbiome. Genome Biol. 2014;15:531. 25517225 10.1186/s13059-014-0531-y PMC4252997

[67] Leung MHY, Tong X, Wilkins D, Cheung HHL, Lee PKH. Volunteer and household attributes influence the dynamics of the personal skin microbiota and its association network. Microbiome. 2018;6:26. 29394957 10.1186/s40168-018-0412-9 PMC5797343

[68] Otberg N, Richter H, Schaefer H, Blume-Peytavi U, Sterry W, Lademann J. Variations of hair follicle size and distribution in different body sites. J Invest Dermatol. 2004;122:14–9. 14962084 10.1046/j.0022-202X.2003.22110.x

[69] Del Rosso JQ, Kircik L. The primary role of sebum in the pathophysiology of acne vulgaris and its therapeutic relevance in acne management. J Dermatol Treat. 2024;35:2296855. 10.1080/09546634.2023.2296855 38146664

[70] Skowron K, Bauza-Kaszewska J, Kraszewska Z, Wiktorczyk-Kapischke N, Grudlewska-Buda K, Kwiecińska-Piróg J, et al. Human skin microbiome: impact of intrinsic and extrinsic factors on skin microbiota. Microorganisms. 2021;9:543. 33808031 10.3390/microorganisms9030543 PMC7998121

[71] Zha Y, Chong H, Yang P, Ning K. Microbial dark matter: from discovery to applications. Gen Proteomics Bioinform. 2022;20:867–81. 10.1016/j.gpb.2022.02.007 PMC10025686 35477055

[72] Dell’Anno A, Danovaro R. Extracellular DNA plays a key role in deep-sea ecosystem functioning. Science. 2005;309:2179. 16195451 10.1126/science.1117475

[73] Jakubovics NS, Shields RC, Rajarajan N, Burgess JG. Life after death: the critical role of extracellular DNA in microbial biofilms. Lett Appl Microbiol. 2013;57:467–75. 23848166 10.1111/lam.12134

[74] Durazzi F, Sala C, Castellani G, Manfreda G, Remondini D, De Cesare A. Comparison between 16S rRNA and shotgun sequencing data for the taxonomic characterization of the gut microbiota. Sci Rep. 2021;11:3030. 33542369 10.1038/s41598-021-82726-y PMC7862389

[75] Hillmann B, Al-Ghalith GA, Shields-Cutler RR, Zhu Q, Gohl DM, Beckman KB, et al. Evaluating the information content of shallow shotgun metagenomics. mSystems. 2018;3:e00069-18. 30443602 10.1128/mSystems.00069-18 PMC6234283

[76] Wang Y, Yan Y, Thompson KN, Bae S, Accorsi EK, Zhang Y, et al. Whole microbial community viability is not quantitatively reflected by propidium monoazide sequencing approach. Microbiome. 2021;9:17. 33478576 10.1186/s40168-020-00961-3 PMC7819323

[77] Coker J, Zhalnina K, Marotz C, Thiruppathy D, Tjuanta M, D’Elia G, et al. A reproducible and tunable synthetic soil microbial community provides new insights into microbial ecology. mSystems. 2022;7:e00951-22. 36472419 10.1128/msystems.00951-22 PMC9765266

[78] Elizaquível P, Sánchez G, Aznar R. Quantitative detection of viable foodborne E. coli O157:H7, Listeria monocytogenes and Salmonel-la in fresh-cut vegetables combining propidium monoazide and real-time PCR. Food Control. 2012;25:704–8.

[79] Hellmann KT, Tuura CE, Fish J, Patel JM, Robinson DA. Viability-resolved metagenomics reveals antagonistic colonization dynamics of Staphylococcus epidermidis strains on preterm infant skin. mSphere. 2021;6:10.1128/msphere.00538-21. 10.1128/mSphere.00538-21 PMC8550141 34523979

[80] Giacomoni PU, Mammone T, Teri M. Gender-linked differences in human skin. J Dermatol Sci. 2009;55:144–9. 19574028 10.1016/j.jdermsci.2009.06.001

[81] Conwill A, Kuan AC, Damerla R, Poret AJ, Baker JS, Tripp AD, et al. Anatomy promotes neutral coexistence of strains in the human skin microbiome. Cell Host Microbe. 2022;30:171-182.e7. 34995483 10.1016/j.chom.2021.12.007 PMC8831475

[82] Higaki S, Morohashi M, Yamagishi T, Hasegawa Y. Comparative study of staphylococci from the skin of atopic dermatitis patients and from healthy subjects. Int J Dermatol. 1999;38:265–9. 10321941 10.1046/j.1365-4362.1999.00686.x

[83] Byrd AL, Deming C, Cassidy SKB, Harrison OJ, Ng W-I, Conlan S, et al. Staphylococcus aureus and Staphylococcus epidermidis strain diversity underlying pediatric atopic dermatitis. Science Translational Medicine. 2017; Available from: https://www.science.org/doi/abs/10.1126/scitranslmed.aal4651. Cited 2021 Sep 7. 10.1126/scitranslmed.aal4651 PMC5706545 28679656

[84] Tanaka A, Cho O, Saito C, Saito M, Tsuboi R, Sugita T. Comprehensive pyrosequencing analysis of the bacterial microbiota of the skin of patients with seborrheic dermatitis. Microbiol Immunol. 2016;60:521–6. 27301664 10.1111/1348-0421.12398

[85] Tsai Y-C, Tsai T-F. Overlapping features of psoriasis and atopic dermatitis: from genetics to immunopathogenesis to phenotypes. Int J Mol Sci. 2022;23:5518. 35628327 10.3390/ijms23105518 PMC9143118

[86] Edslev SM, Agner T, Andersen PS. Skin microbiome in atopic dermatitis. Acta Derm Venereol. 2020;100:5769. 32419029 10.2340/00015555-3514 PMC9189751

