# Supplementary material for: Absolute quantification of the living skin microbiome overcomes relic-DNA bias and reveals specific patterns across volunteers
Source: Microbiome. 2025 Mar 4;13:65. doi: 10.1186/s40168-025-02063-4 (PMC11877739; doi:10.1186/s40168-025-02063-4)
Supplement: Supplementary file 2 — Supplementary Material 1: Figure S1. Mapping parameters and relative abundance of all samples sequenced part of the study. a) Box plot of percentage of reads mapped to the reference human genome (GRCh38.p14) after quality and swab + blank control filtering. b) Bar plot of average total reads per sample that passed quality filtering for swab/blank filtering, then to host genome filtering, and then for mapping to the database and the reads that mapped (bacterial genomes only in SMGC). c) Box plot of percentage of reads mapped to the reference database (SMGC) after quality, swab and blank and human filtering. d) Density plot of the ratio of the reference genome in SMGC database covered in all samples in the dataset, which was used to constrain alignment and read assignment using Zebra. Figure S2: Principal component analysis of Raw and PMA-treated samples. a) PCA of Raw samples. b) PCA of PMA-treated samples. c) Relative abundance bar plots of identified taxa across all samples sequenced as part of the study. Identified species with median RPKM > 0.05% reported. Figure S3: PMA index and alpha diversity index shows no significant change between Raw and PMA-treated samples. a) Heatmap of the PMA_index (formula above) computed on top taxa identified across all samples averaged by body site (FH: forehead, UB: upper back, AC: antecubital crease, PC: popliteal crease, FA: forearm, Abd: abdomen). Heatmaps are shaded by the PMA-index score, where a score greater than 0.5 indicates an underestimation of that taxa in samples, and lesser than 0.5 suggests an overestimation of that taxa in samples. Inset values represent the standard deviation in the computed PMA_index across individuals and the paired Wilcoxon test with multiple testing“fdr” corrections computed for each taxa by body site : *** p<0.005, ** p<0.01, * p<0.05, ns non-significant. Body Site acronyms: FH (forehead), UB (upper back), AC (antecubital crease), PC (popliteal crease), FA (forearm), Abd (abdomen). Figure S4 [file 40168_2025_2063_MOESM1_ESM.docx]

### **Supplemental Information**

**Absolute Quantification of the Living Skin Microbiome Overcomes relic DNA Bias and Reveals Site-Specific Patterns Across Individuals**

Deepan Thiruppathy^✝1^, Oriane Moyne^✝2^, Clarisse Marotz^2^, Michael Williams^2^, Perris Navarro^2^, Livia Zaramela^2^, Karsten Zengler^1,2,3,4^*

**
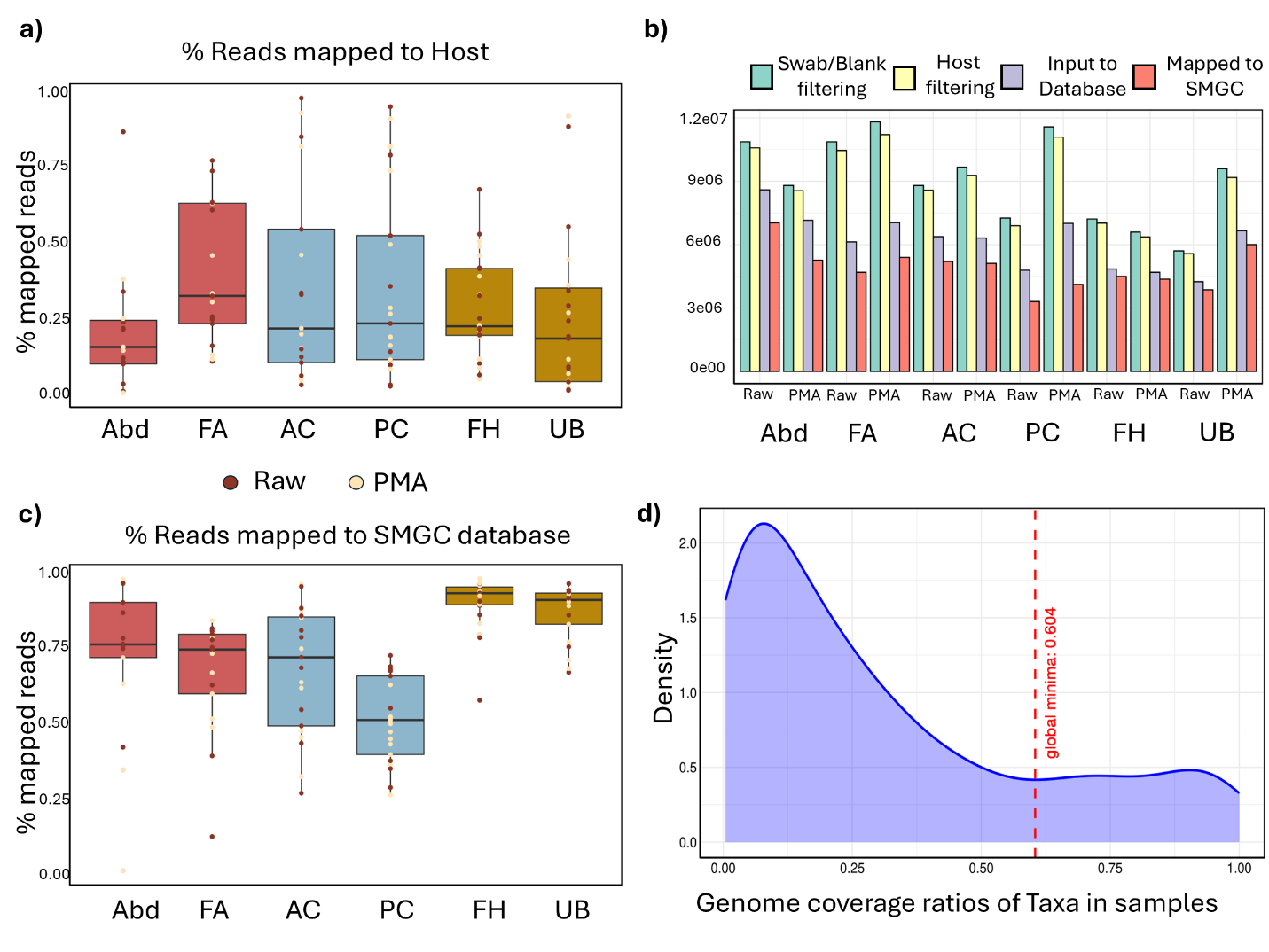
**

**Figure S1: Mapping parameters and relative abundance of all samples sequenced part of the study.**

a) Box plot of percentage of reads mapped to the reference human genome (GRCh38.p14) after quality and swab + blank control filtering.
b) Bar plot of average total reads per sample that passed quality filtering for swab/blank filtering, then to host genome filtering, and then for mapping to the database and the reads that mapped (bacterial genomes only in SMGC).

c) Box plot of percentage of reads mapped to the reference database (SMGC) after quality, swab and blank and human filtering.

d) Density plot of the ratio of the reference genome in SMGC database covered in all samples in the dataset, which was used to constrain alignment and read assignment using Zebra.

**
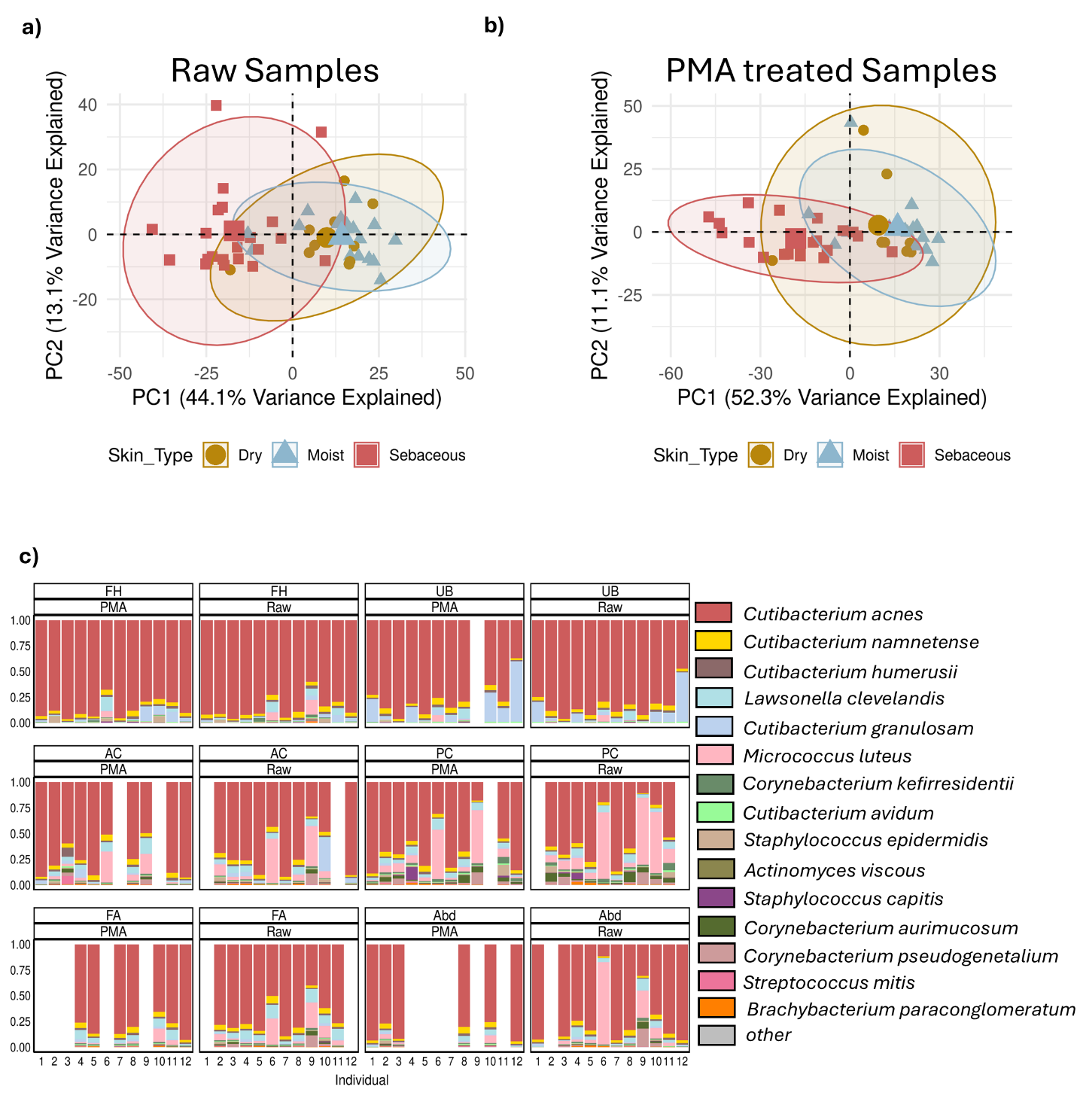
**

**Figure S2: Principal component analysis of Raw and PMA-treated samples.**a) PCA of Raw samples

b) PCA of PMA-treated samples

c) Relative abundance bar plots of identified taxa across all samples sequenced as part of the study. Identified species with median RPKM > 0.05% reported.

**
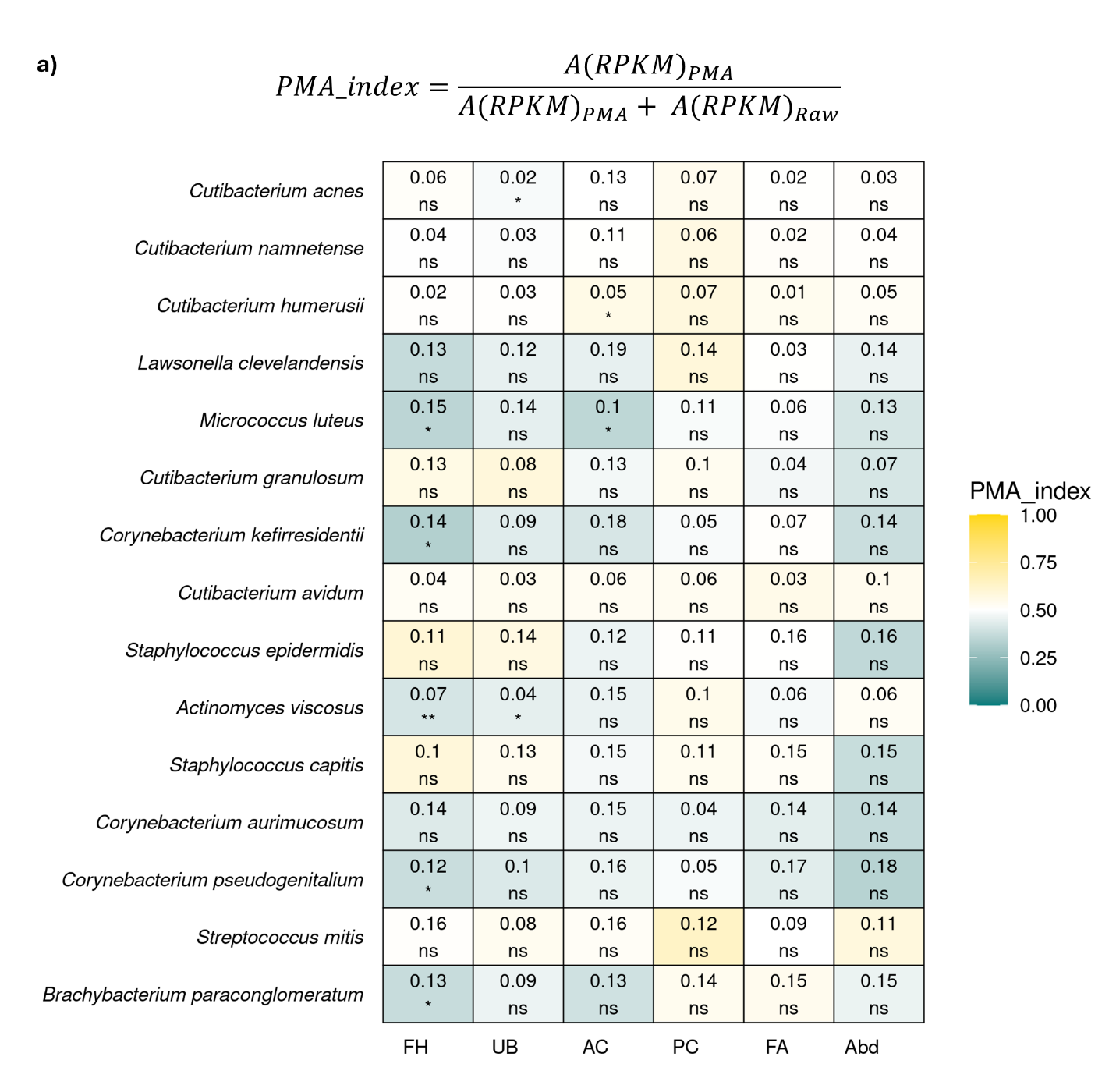
**

**Figure S3: PMA index and alpha diversity index shows no significant change between Raw and PMA-treated samples.**

a) Heatmap of the PMA_index (formula above) computed on top taxa identified across all samples averaged by body site (FH: forehead, UB: upper back, AC: antecubital crease, PC: popliteal crease, FA: forearm, Abd: abdomen). Heatmaps are shaded by the PMA-index score, where a score greater than 0.5 indicates an underestimation of that taxa in samples, and lesser than 0.5 suggests an overestimation of that taxa in samples. Inset values represent the standard deviation in the computed PMA_index across individuals and the paired Wilcoxon test with multiple testing“fdr” corrections computed for each taxa by body site : *** *p*<0.005, ** *p*<0.01, * *p<*0.05, ns non-significant. Body Site acronyms: FH (forehead), UB (upper back), AC (antecubital crease), PC (popliteal crease), FA (forearm), Abd (abdomen)

**
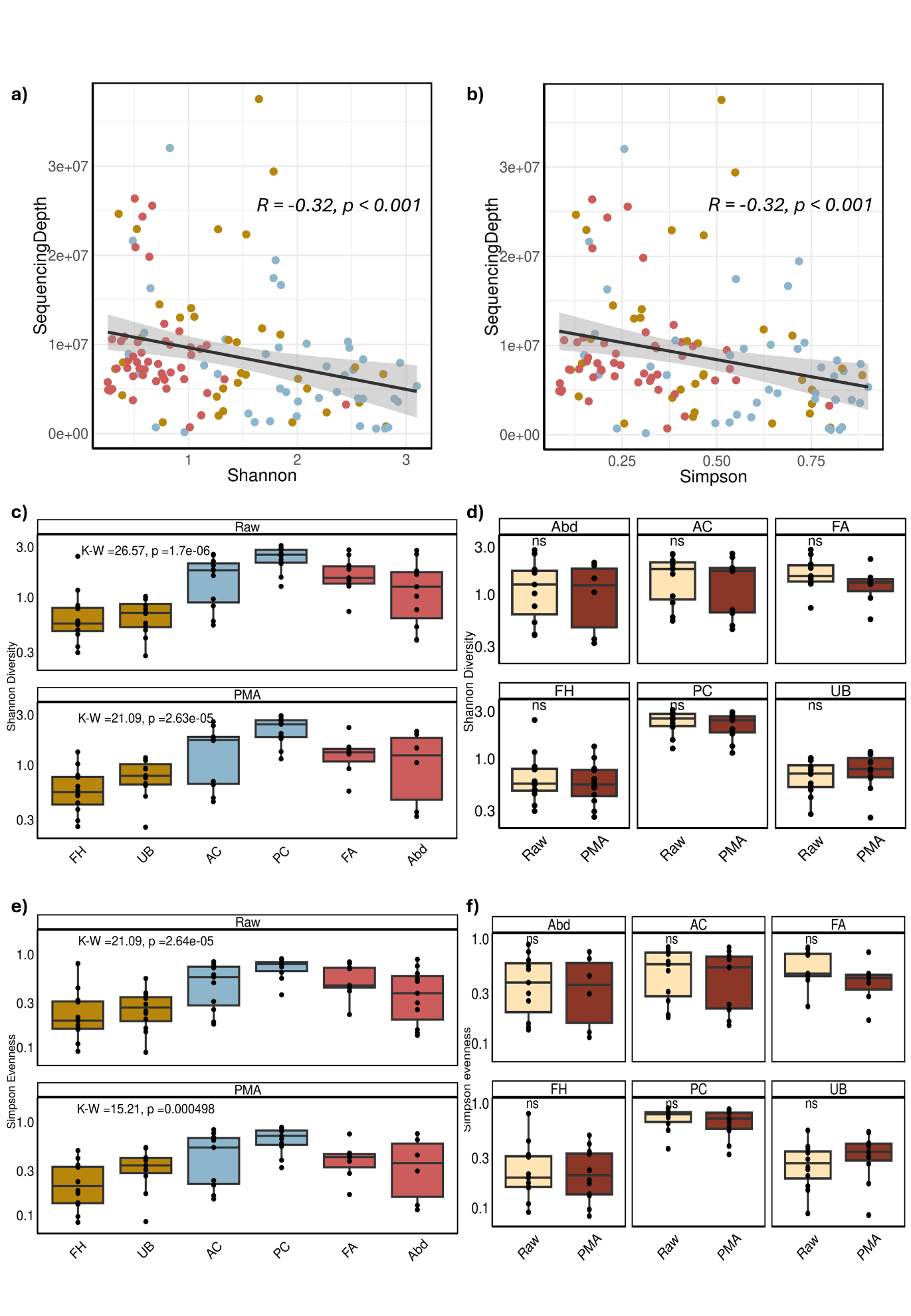
**

**Figure S4: Alpha diversity of samples reveals no significant bias from relic-DNA in skin microbiome samples.**

a) Correlation plots between sequencing depth of all samples (library size) and a) Shannon diversity or b) Simpson evenness alpha diversity metrics. Spearman correlation of -0.323 for both a) and b), suggesting no effect from sequencing depth on the computed alpha diversity.

c) Box plot of Shannon diversity of all samples in Raw vs PMA-treated samples grouped by body site and colored by skin type. Both Raw and PMA-treated samples retain similar patterns for cross skin-type diversities. (Kruskal–Wallis multi-group non-parametric test computed on Skin Type, n = 57 (PMA) and n = 66 (Raw)).

d) Box plot of Shannon diversity comparisons between samples from the same body site between raw and PMA treatments shows no significant difference (Wilcoxon rank-sum test, multiple comparisons “fdr” correction).

e) Box plot of Simpson evenness of all samples in Raw vs PMA-treated samples grouped by body site and colored by skin type. Both Raw and PMA-treated samples retain similar patterns for cross skin-type diversities. (Kruskal–Wallis multi-group non-parametric test computed on Skin Type, n = 57 (PMA) and n = 66 (Raw)).

f) Box plot of Simpson evenness comparisons between samples from the same body site between raw and PMA treatments shows no significant difference (Wilcoxon rank-sum test, multiple comparisons “fdr” correction).

**
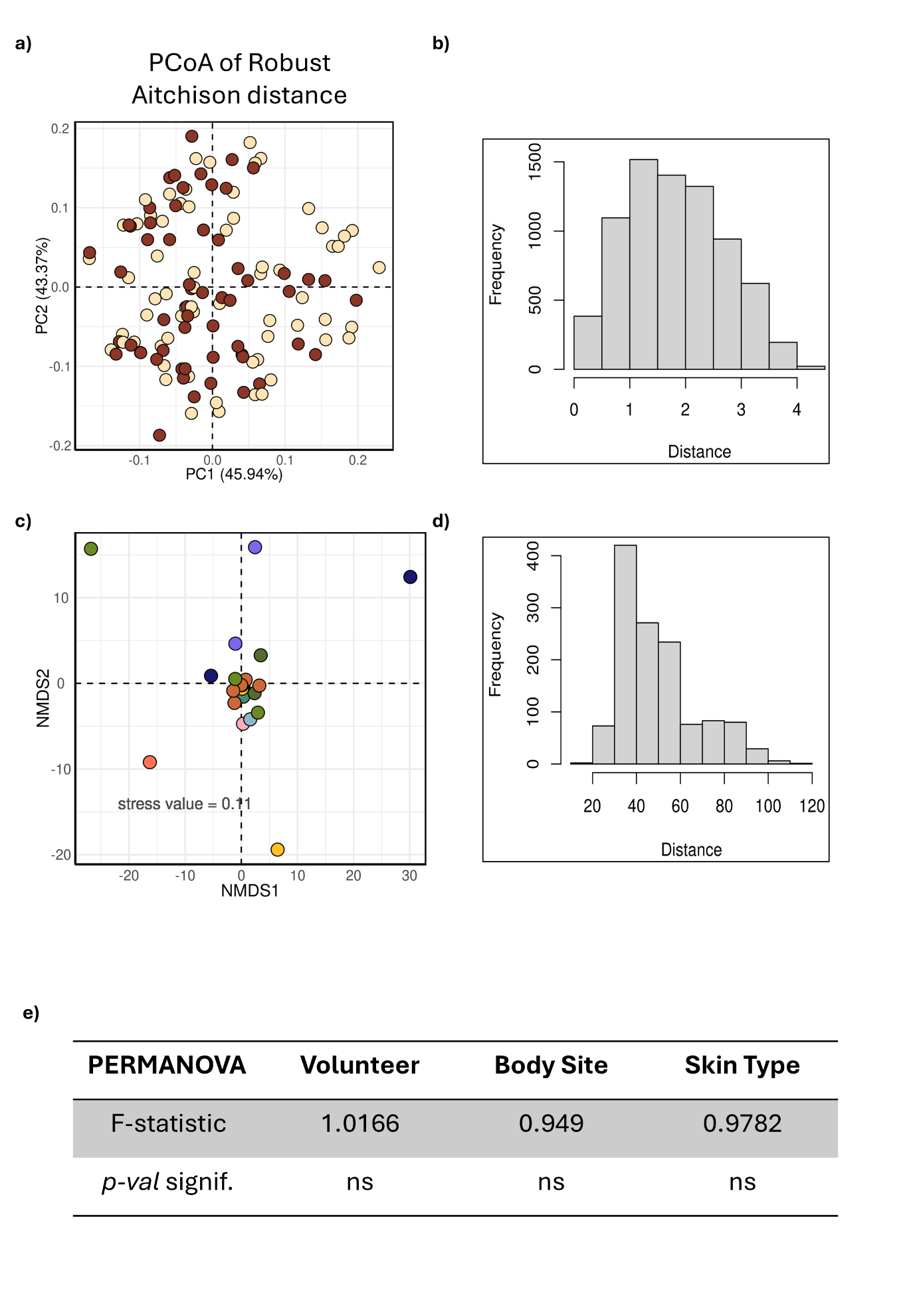
**

**Figure S5: relic-DNA in samples is not personalized.**

a) PCoA (Principal Coordinate Analysis) on the robust aitchison distances between all samples (n = 123) in the dataset, colored by treatment.

b) Histogram plot of the robust aitchison distances between all samples (raw + PMA).

c) NMDS plot of the robust aitchison distances between the relative abundance of the relic-DNA proportion only in all samples aligned to Web Of Life database (Zhu et al. 2019) colored by volunteer.

d) Histogram plot of the robust aitchison distances between samples calculated using only the relic-DNA portion of the samples.

e) PERMANOVA results computed on robust aitchison distances between the relic-DNA proportion in samples only to test grouping by Volunteer, Body Site or Skin Type. (*p* values >0.05, ns (not significant))

**
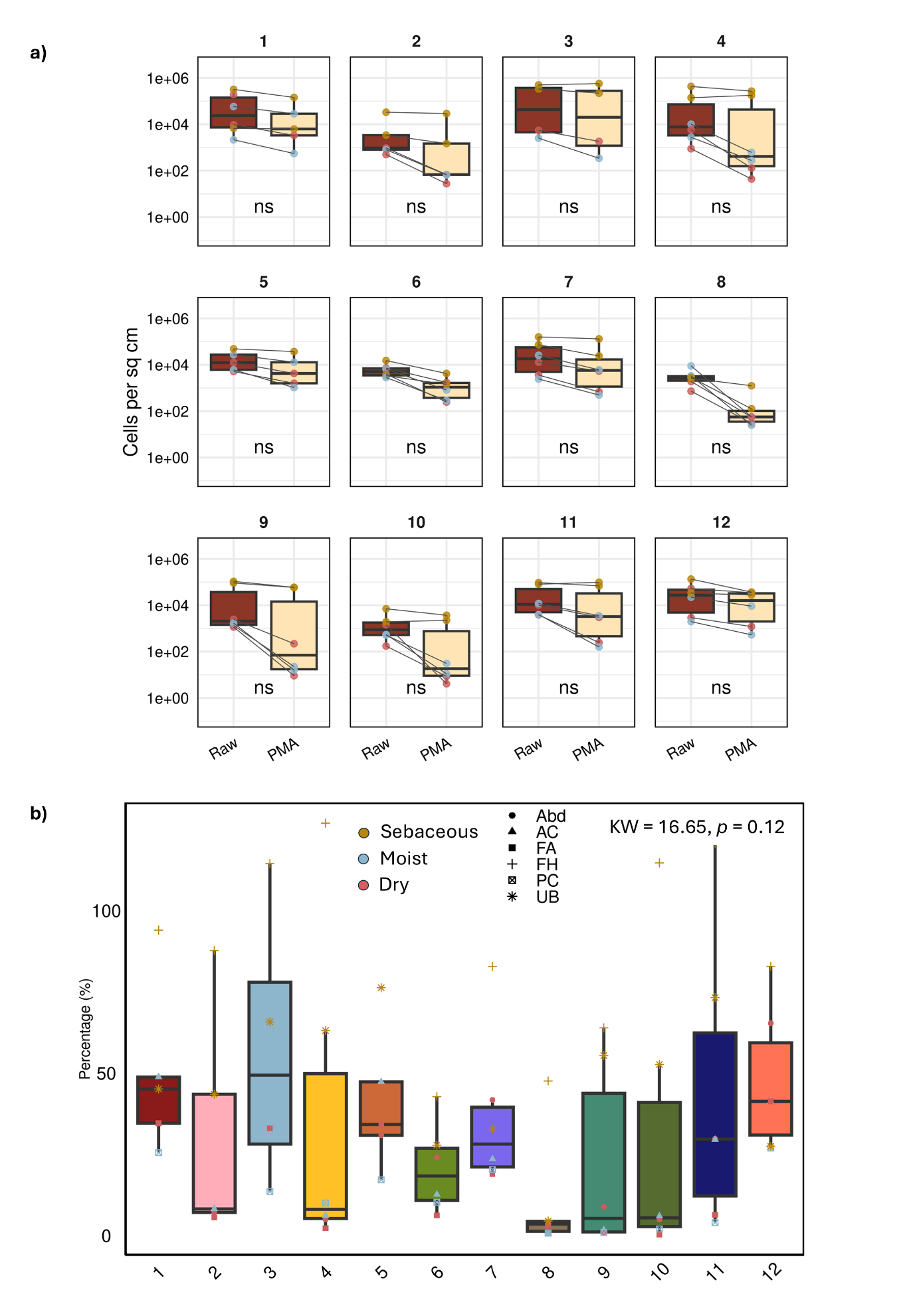
**

**Figure S6: Comparison of total cells vs live cells and percentage live across all volunteers.**

a) Box plot of cell per sq cm enumerated from all body sites in an volunteer indicates no volunteer had a significant drop in cell counts between treatments. (Wilcoxon paired test, FDR multiple test corrections. *P >* 0.05 ns)

b) Live fraction (%) computed across all samples from an volunteer (Kruskal-Wallis multiple group comparisons test and Dunn’s pairwise comparison).

**
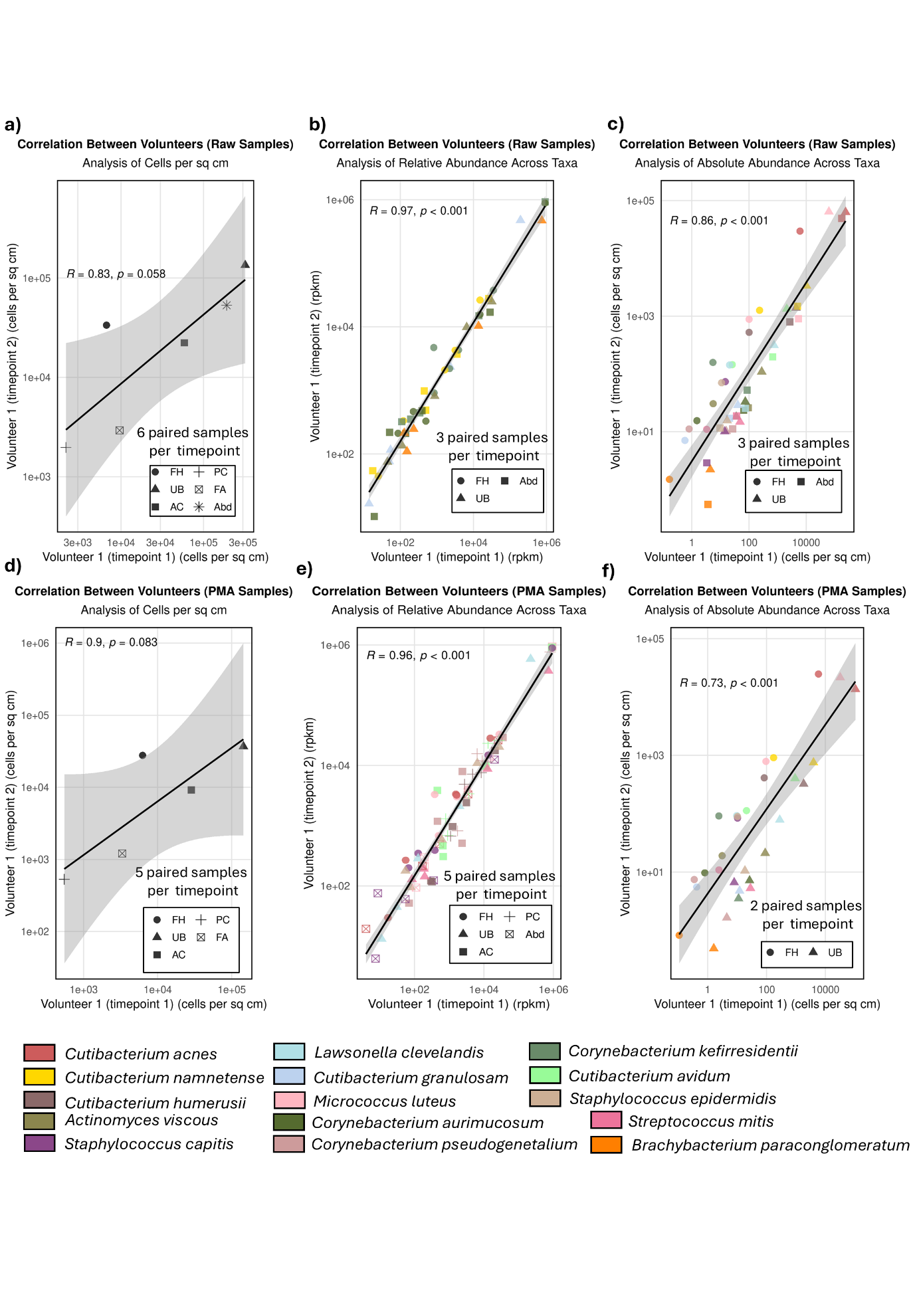
**

**Figure S6: Comparison of total cells vs live cells and percentage live across all volunteers.**

a) Box plot of cell per sq cm enumerated from all body sites in a volunteer indicates no volunteer had a significant drop in cell counts between treatments. (Wilcoxon paired test, FDR multiple test corrections. *P >* 0.05 ns)

b) Live fraction (%) computed across all samples from a volunteer (Kruskal-Wallis multiple group comparisons test and Dunn’s pairwise comparison).

**
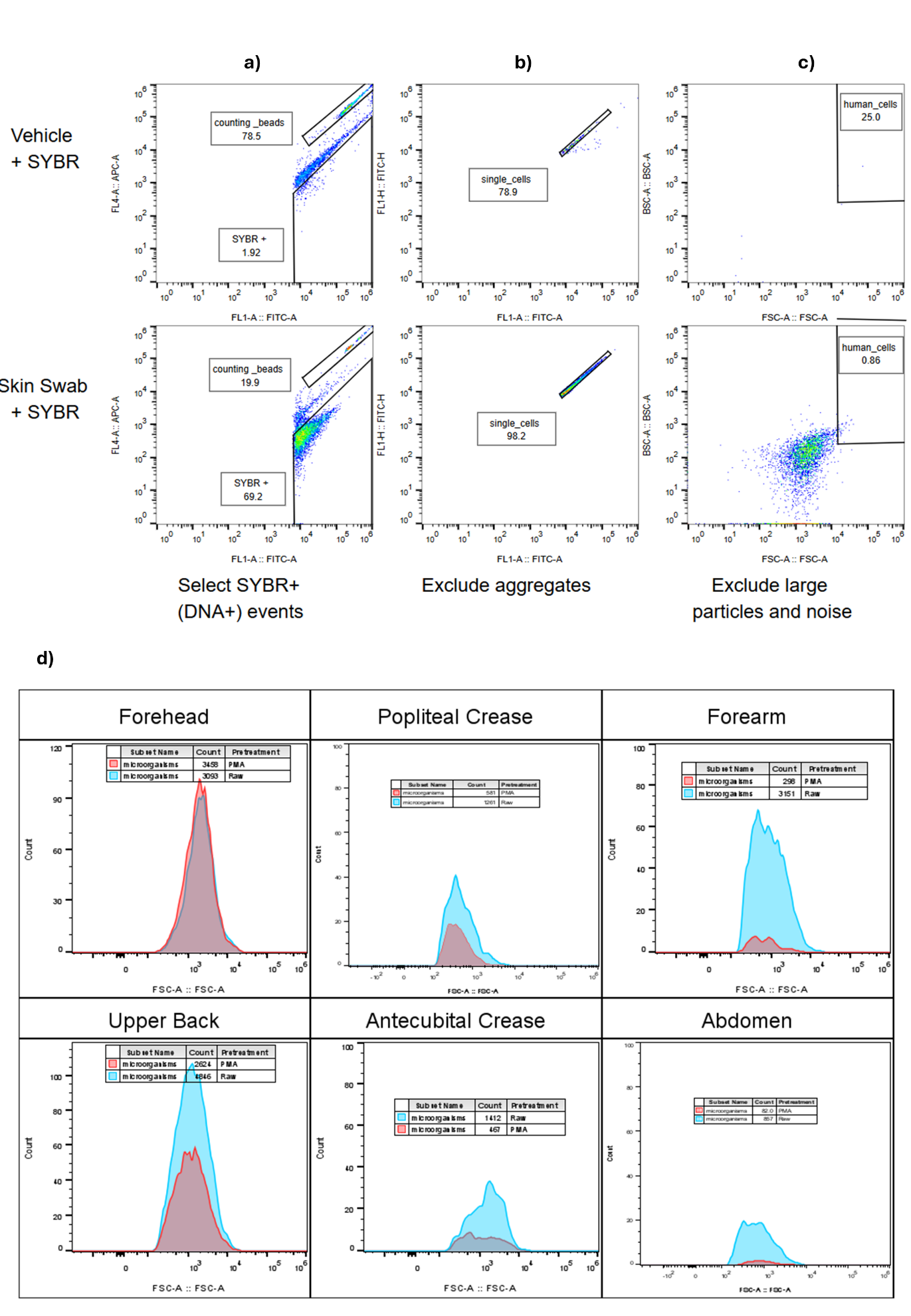
**

**Figure S8: Representative example of the gating strategy applied to all flow cytometry samples.**

Flow cytometry gating strategy adapted from Marotz et al. and Props et al. Skin swab samples collected in 1X PBS were vortexed at maximum speed. 400 uL of this was filtered across a 5-μm filter to remove human cells and swab lint, stained with SYBR green (0.1X final), and processed on a Sony SH800 instrument with Spherotech counting beads. The threshold was set on the FL1 detector.

a) The first gate selects for events with enhanced 525-nm specific emission to select DNA-positive events.

b) Doublets were excluded by selecting only events following a linear trend between FL1 height and area.

c) Human cells are excluded by their large size on forward (FSC-A) and side (SSC-A) scatter area.

d) Representative example of cell counts obtained from Raw (blue) vs PMA-treatd (red) samples across the body sites sampled using the gating strategy.

**
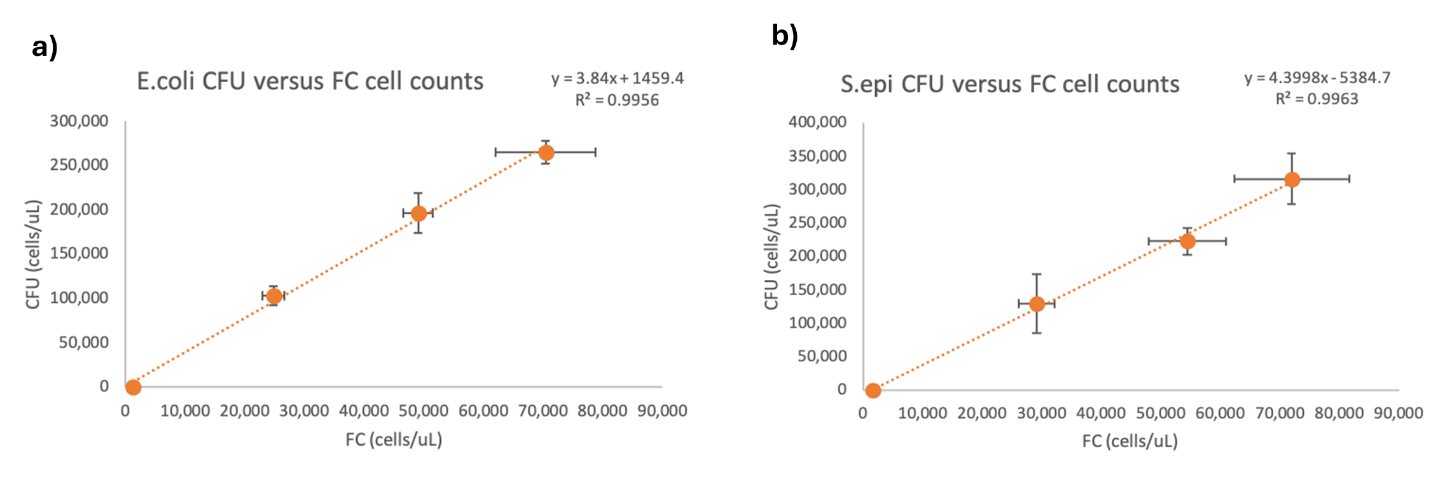
**

**Figure S9: Correlation between an overnight control of (a) *Escherichia coli* and (b) *S. epidermidis* CFU counts and counts estimated from flow cytometry of PMA-treated aliquots of the cultures.**

**Table S1: Metadata of individuals involved in the study.**

| **ID** | **Age** | **Sex** | **Ethnicity** | **Face** | | | | **Body Shower** | | **Body Care** | | **Exercise/ week** | **Ocean /week** | **Pool /week** | **Sauna/Hammam/week** | **Pets** | **Comments** |
| --- | --- | --- | --- | --- | --- | --- | --- | --- | --- | --- | --- | --- | --- | --- | --- | --- | --- |
|  |  |  |  | **Method** | **Freq (per week)** | **Product** | **Sunscreen (per week)** | **Product** | **Freq** | **Product** | **Freq** |  |  |  |  |  |  |
| 1 | 33 | F | white | water | 1 | natural | 1 | bar soap | 0.5 | none | 0 | 7 | 3 | 0 | 0 | cats |  |
| 2 | 34 | F | latino | makeup remover + water + soap + lotion | 2 | industrial | 0 | bar soap | 1 | industrial | 2 | 4 | 0 | 0 | 0 | none |  |
| 3 | 33 | M | indian | water | 1 | industrial | 1 | body wash gel | 1 | industrial | 1 | 3 | 0 | 0 | 0 | none |  |
| 4 | 29 | M | white | soap | 0.5 | natural | 0.5 | bar soap | 1 | none | 0 | 7 | 3 | 0 | 0 | none |  |
| 5 | 28 | F | white | soap | 0 | none | 0.5 | bar soap | 1.5 | none | 0 | 10 | 2 | 2 | 0 | none |  |
| 6 | 23 | M | indian | soap | 0 | none | 0 | body wash gel | 1 | none | 0 | 4 | 0 | 0 | 0 | cat |  |
| 7 | 34 | F | latino | Soap + lotion | 1 | industrial | 1 | bar soap | 2 | industrial | 2 | 5 | 2 | 2 | 0 | none |  |
| 8 | 29 | F | white | soap | 2 | natural | 0 | bar soap | 1 | Industrial / natural | 0.5 | 1 | 0 | 0 | 0 | none | 3 months pregnant |
| 9 | 37 | M | indian | water | 0.5 | industrial | 0 | body wash gel | 1.5 | none | 0 | 7 | 0 | 0 | 0 | none |  |
| 10 | 52 | M | white | water | 0 | none | 0 | body wash gel | 1 | none | 0 | 2 | 0 | 1 | 0 | dog |  |
| 11 | 20 | F | asian | makeup remover + water + soap + lotion | 1 | industrial | 0 | bar soap | 1 | industrial | 1 | 3 | 0 | 0 | 0 | none | daily acne spa treatment face |
| 12 | 33 | F | white | water | 1 | natural | 1 | bar soap | 0.5 | none | 0 | 7 | 3 | 0 | 0 | cats | Repeated sample from Volunteer 1 |

**Table S2: Body site metadata and dimensions of plastic patterns.**

| **ID** | **Body Site** | **Skin Type** | **Dimensions** | | |
| --- | --- | --- | --- | --- | --- |
|  |  |  | **Width (cm)** | **Height (cm)** | **Area (cm^2^)** |
| FH | Forehead | sebaceous | 7.80 | 5.60 | 43.68 |
| UB | Upper back | sebaceous | 9.70 | 6.50 | 63.05 |
| AC | Antecubital crease | moist | 7.00 | 5.00 | 35 |
| PC | popliteal crease | moist | 8.30 | 6.10 | 50.63 |
| FA | Forearm | dry | 11.40 | 4.50 | 51.3 |
| Abd | Abdomen | dry | 7.10 | 7.10 | 50.41 |
